# Efficient purification of ethene by an ethane-trapping metal-organic framework

**DOI:** 10.1038/ncomms9697

**Published:** 2015-10-29

**Authors:** Pei-Qin Liao, Wei-Xiong Zhang, Jie-Peng Zhang, Xiao-Ming Chen

**Affiliations:** 1 MOE Key Laboratory of Bioinorganic and Synthetic Chemistry, School of Chemistry and Chemical Engineering, Sun Yat-Sen University, Guangzhou 510275, P.R. China

## Abstract

Separating ethene (C_2_H_4_) from ethane (C_2_H_6_) is of paramount importance and difficulty. Here we show that C_2_H_4_ can be efficiently purified by trapping the inert C_2_H_6_ in a judiciously designed metal-organic framework. Under ambient conditions, passing a typical cracked gas mixture (15:1 C_2_H_4_/C_2_H_6_) through 1 litre of this C_2_H_6_ selective adsorbent directly produces 56 litres of C_2_H_4_ with 99.95%+ purity (required by the C_2_H_4_ polymerization reactor) at the outlet, with a single breakthrough operation, while other C_2_H_6_ selective materials can only produce *ca*. ⩽ litre, and conventional C_2_H_4_ selective adsorbents require at least four adsorption–desorption cycles to achieve the same C_2_H_4_ purity. Single-crystal X-ray diffraction and computational simulation studies showed that the exceptional C_2_H_6_ selectivity arises from the proper positioning of multiple electronegative and electropositive functional groups on the ultramicroporous pore surface, which form multiple C–H···N hydrogen bonds with C_2_H_6_ instead of the more polar competitor C_2_H_4_.

## Abstract (web summary)

The separation of high purity ethene from the mixed gaseous products of cracking poses significant obstacles. Here, the authors present a metal-organic framework which, in contrast to most absorbents, selectively binds the less polar ethane thus allowing the efficient collection of the target product.

## Graphical abstract

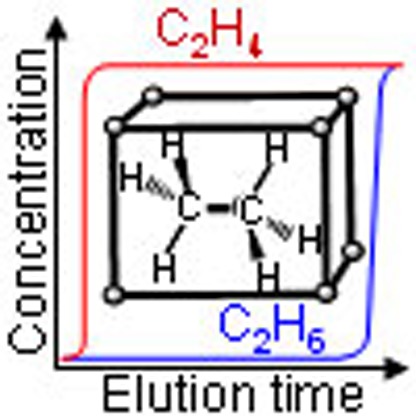

As the most important chemical product, ethene (C_2_H_4_) is generally obtained through steam cracking and thermal decomposition of naphtha or ethane (C_2_H_6_) (ref. 1). Besides being obtained as a byproduct of petroleum refining, C_2_H_6_ is also isolated on an industrial scale from natural gas (CH_4_ 70∼96%, C_2_H_6_ 1∼14% and CO_2_ 0∼8%) (ref. 2). As a result of their similar physical properties, it is difficult to separate C_2_H_6_, C_2_H_4_ and CO_2_ (refs 3, 4, 5). In industry, C_2_H_6_ and C_2_H_4_ are separated by cryogenic high-pressure distillation, typically at 7–28 bar and 183–258 K using very high towers consisting of over 150 trays, which is very energy consuming (7 GJ t^−1^) and constitutes a notable portion of the ethylene cost^6^,^7^. To save energy, separation methods effective at the ambient temperature and pressure are highly demanded^8^,^9^,^10^,^11^. Passing the gas mixture through a fixed-bed adsorber can be a very simple and promising approach to afford low energy consumption and high product purity.

Because unsaturated hydrocarbons like to coordinate with metal ions, C_2_H_4_ can be selectively bound and separated from its saturated counterpart C_2_H_6_ (refs 12, 13, 14, 15, 16). Compared with other types of porous materials, porous metal-organic frameworks (MOFs) are unique for their diversified/designable framework structures and pore surfaces, including the ease of introducing open metal sites (OMSs), which have shown great potentials for C_2_H_4_/C_2_H_6_ separation^17^,^18^,^19^,^20^,^21^,^22^. In the fix-bed separation process, the un-adsorbed C_2_H_6_ first breakthrough, and C_2_H_4_ enriched in the stationary phase is later obtained by heating and/or inert-gas blowing. Because the un-adsorbed C_2_H_6_ residing in the mobile phase contaminates the desired product C_2_H_4_ during the desorption stage, the highest C_2_H_4_ purity produced by a full adsorption–desorption cycle can just reach 99%+ (refs 13, 17, 23, 24), and at least four such cycles are necessary to achieve 99.95%+ (ref. 25), the lower limit required by the C_2_H_4_ polymerization reactor^26^,^27^,^28^. Obviously, this problem can be solved by using a C_2_H_6_ selective adsorbent, which not only improves the C_2_H_4_ purity but also reduces energy consumption. The simple separation operation and device (just one adsorption process in a single breakthrough operation) are also necessary for onsite supply of purified C_2_H_4_. However, such an unusual adsorption behaviour has been only reported for a few low-polarity or hydrophobic MOFs^29^,^30^,^31^,^32^,^33^,^34^,^35^,^36^, and their C_2_H_4_/C_2_H_6_ separation performances (that is, C_2_H_6_/C_2_H_4_ selectivities) are poor, because the polarities of C_2_H_4_ and C_2_H_6_ are very similar and can be hardly distinguished by hydrophobic adsorbents.

As C_2_H_6_ possesses the lowest polarity (quadrupole moment) compared with similar molecules such as C_2_H_4_ and CO_2_ (Supplementary Table 1)^37^; polar adsorbents are generally selective for the latter gases. However, considering that the electropositive and electronegative portions locate quite differently among these gas molecules, we speculated that by rational utilization of polar functional groups, it is still possible to design a MOF with optimized pore size/shape and surface electrostatic distribution that can bind C_2_H_6_ much stronger than for C_2_H_4_. Herein, we report the design, structure and gas adsorption/separation properties of such a C_2_H_6_-trapping MOF, which is useful for not only direct producing highly pure C_2_H_4_ from C_2_H_4_/C_2_H_6_ mixtures, but also efficient separation of four-component CH_4_/C_2_H_4_/C_2_H_6_/CO_2_ mixtures and extraction of C_2_H_6_ from natural gas.

## Results

### Synthesis, structure and stability

**Figure 1:**
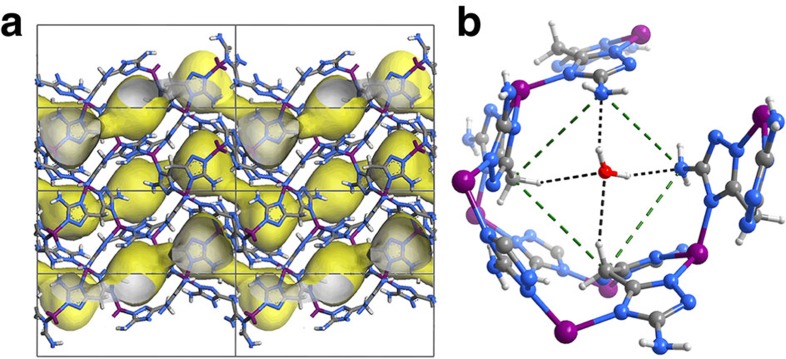
X-ray crystal structure of MAF-49·H_2_O. (**a**) Framework (Zn purple, C dark grey, H light grey, N blue) and pore surface (yellow/grey curved surface) structures. Guest molecules are omitted for clarity. (**b**) Local environment and hydrogen-bonding interactions of the narrowest channel neck (highlighted by green dashed lines).

Bis(5-amino-1*H*-1,2,4-triazol-3-yl)methane (H_2_batz) with two 3-amino-1,2,4-triazole rings bridged by a methylene group was designed as a new ligand combining multiple nitrogen atoms as hydrogen-bonding acceptors and methylene groups as dipole repulsion groups, as well as short bridging lengths for construction of an ultramicroporous framework. Reaction of H_2_batz and Zn(OH)_2_ in dilute aqueous ammonia produced a porous metal-azolate framework [Zn(batz)]·0.5H_2_O (MAF-49·H_2_O). Single-crystal X-ray diffraction (SCXRD) analysis of MAF-49·H_2_O (Supplementary Table 2 and Supplementary Data 1) showed that each Zn(II) is tetrahedrally coordinated by four triazolate nitrogen atoms from three batz^2–^ ligands (Supplementary Fig. 1), and each batz^2–^ ligand coordinates to three Zn(II) ions in a bisimidazolate mode, giving a three-dimensional (3D) coordination framework with narrow 1D zigzag channels (Fig. 1a). Since only four of the eight nitrogen donors of batz^2–^ are utilized according to the coordination requirement of Zn(II), the pore surface of MAF-49 is rich with electronegative nitrogen atoms, although some of them form intra-framework N–H···N hydrogen bonds to reduce their abilities as hydrogen-bonding acceptors. Notably, the narrowest section of the 1D channel (3.3 × 3.0 Å^2^) is approximately a folded four-membered ring defined by a pair of free amino groups (with their lone electron pairs) and a pair of methylene groups with a *cis*-configuration, which is occupied by a guest H_2_O molecule with two O–H···N and two C–H···O hydrogen bonds (Fig. 1b).

Thermogravimetry and powder XRD showed that MAF-49·H_2_O can be readily activated and is stable to 450 °C in nitrogen (Supplementary Fig. 2), in boiling water for at least 1 month and in aqueous acid/base (4≤pH≤12) at room temperature for at least 1 week (Supplementary Fig. 3), which is extraordinary among MOFs and can be partly explained by the strong metal-azolate coordination bonds^38^. SCXRD showed that complete dehydration leads to a slight framework expansion (0.17% in volume, Supplementary Table 2 and Supplementary Data 2).

### Gas adsorption property and mechanism

**Figure 2:**
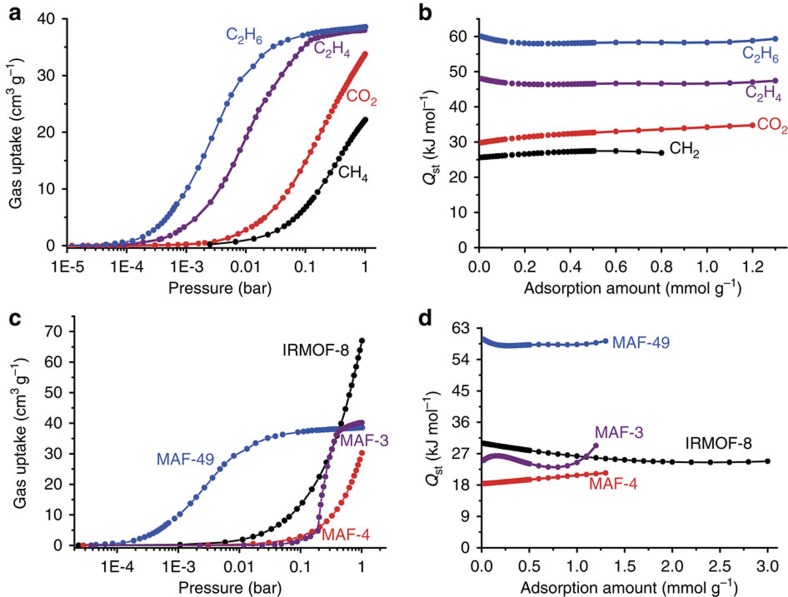
Single-component gas adsorption properties. (**a**) Gas adsorption isotherms for C_2_H_6_, C_2_H_4_, CO_2_ and CH_4_ in MAF-49 at 316 K. (**b**) The coverage-dependent C_2_H_6_, C_2_H_4_, CO_2_ and CH_4_ adsorption enthalpy obtained by the Virial method. (**c**) C_2_H_6_ adsorption isotherms of MAF-49, MAF-3, MAF-4 and IRMOF-8 measured at 316 K. (**d**) Coverage-dependent C_2_H_6_ adsorption enthalpy of MAF-49, MAF-3, MAF-4 and IRMOF-8.

Single-component adsorption isotherms for CH_4_, C_2_H_6_, C_2_H_4_ and CO_2_ were measured for guest-free MAF-49 at 298 K, 307 K and 316 K (Fig. 2a and Supplementary Fig. 4). According to their different isotherm shapes, it can be judged that the host–guest binding follows C_2_H_6_>C_2_H_4_>CO_2_>CH_4_. The gas adsorption enthalpies were calculated quantitatively by Virial analyses (Fig. 2b and Supplementary Fig. 5), which are 60 kJ mol^−1^, 48 kJ mol^−1^, 30 kJ mol^−1^ and 25 kJ mol^−1^ for C_2_H_6_, C_2_H_4_, CO_2_, and CH_4_, respectively, at zero-coverage. The mixed gas adsorption isotherms for equimolar C_2_H_6_/C_2_H_4_, C_2_H_6_/CO_2_ and C_2_H_6_/CH_4_ mixtures were simulated by the ideal adsorbed solution theory^39^, in which the single-component adsorption isotherms were fitted by the Langmuir−Freundlich model (Supplementary Fig. 6). At total pressure of 100 kPa and a temperature of 316 K, the C_2_H_6_/C_2_H_4_, C_2_H_6_/CO_2_ and C_2_H_6_/CH_4_ selectivities of these mixtures were calculated as *ca*. 9, 40 and 170, respectively (Supplementary Fig. 7). Notably, the C_2_H_6_/C_2_H_4_ selectivity of MAF-49 is much higher than the highest value in the literature (2.4 for IRMOF-8 at 318 K) (ref. 31). Except for CH_4_ with obviously lower molecular weight and boiling point, which interacts weakly with all adsorbents, the binding strength order of MAF-49 for other three heavier gases is unusual. Among a variety of physical properties of the four gases, only the polarizability trend is consistent with the binding trend (Supplementary Table 1). Nevertheless, the small differences of their polarizabilities are not enough to explain the large variation of their adsorption enthalpies, especially for C_2_H_6_ and C_2_H_4_. Notably, the C_2_H_6_ adsorption enthalpy is significantly higher than reported values, while the C_2_H_4_ one is moderate^18^.

To elucidate the very different C_2_H_6_, C_2_H_4_ and CO_2_ affinities of MAF-49, their preferential host–guest structures and energy changes were calculated by grand canonical Monte Carlo simulation and further periodic density functional theory optimization. The obtained binding energies of the final host–guest structures are −56.7, −45.5 and −41.3 kJ mol^–1^ for C_2_H_6_, C_2_H_4_ and CO_2_, respectively. However, to adsorb these gas molecules, the host framework undergoes different structural distortions from the guest-free form and consumes energies of +0.2 kJ mol^–1^, +0.3 kJ mol^–1^ and +5.6 kJ mol^–1^, respectively. Taking both the host–guest binding and host-framework distortion into consideration, the total energies or adsorption enthalpies can be calculated as −56.5 kJ mol^–1^, −45.2 kJ mol^–1^ and −35.7 kJ mol^–1^ for C_2_H_6_, C_2_H_4_, and CO_2_, respectively, which are consistent with the experimental values (Supplementary Table 3). In the density functional theory optimized host–guest structures, it can be seen that C_2_H_6_, C_2_H_4_ and CO_2_ are all adsorbed in or very close to the narrowest channel neck, but they interact very differently with the pore surface.

**Figure 3:**
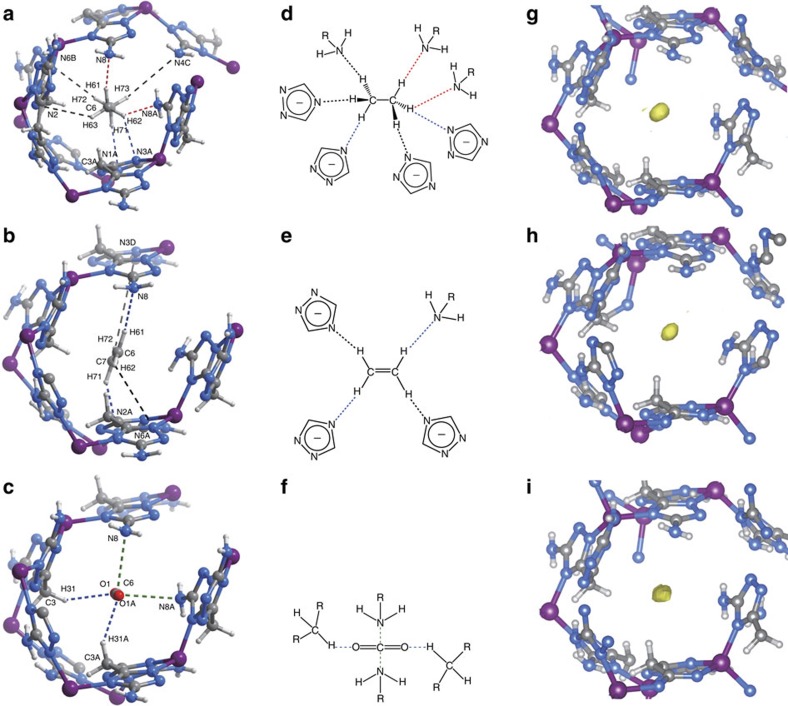
Host–guest fittings and interactions. Preferential adsorption sites for (**a**) C_2_H_6_, (**b**) C_2_H_4_ and (**c**) CO_2_ in MAF-49 revealed by computational simulations (Zn purple, C dark grey, H light grey, N blue). Schematic representation of the corresponding host–guest interactions for (**d**) C_2_H_6_, (**e**) C_2_H_4_ and (**f**) CO_2_. Strong (H···N/O<2.3 Å), weak (2.3 Å<H···N/O<2.8 Å) and almost negligible (H···N/O>2.8 Å) C–H···N interactions are displayed as red, blue and black dashed lines, respectively. 3D electron density maps (*F*_o_–*F*_c_ contoured at 0.80 e Å^−3^ in yellow) of MAF-49 loaded with trace amounts of (**g**) C_2_H_6_, (**h**) C_2_H_4_ and (**i**) CO_2_.

C_2_H_6_ forms three strong C–H···N hydrogen bonds and three weak C–H···N electrostatic interactions with MAF-49 (Fig. 3a,d, Supplementary Table 4). Specifically, one methyl group interacts with two amino groups and an coordinated triazolate nitrogen atom of the narrowest channel neck, forming one very short and directional (C6-H61···N8) and one unsymmetrical-bifurcated/three-centred (C6-H62···N8A/N3A) hydrogen bonds, in which the H···N separations (2.15 Å) are much shorter than the sum of van der Waals radii of nitrogen (1.55 Å) and hydrogen (1.20 Å) atoms. The third strong hydrogen bond involves the hydrogen atom (H71) of another methyl group and a coordinated triazolate nitrogen atom (C8-H71···N1A), which is approximately centro-symmetric with the strongest one (C6-H61···N8) about the molecular centre and fits well with the most stable stagger conformation of C_2_H_6_. Besides, the less polar part of the pore surface, that is, two methylene groups of the batz^2–^ ligand (C3), fits well with the guest C_2_H_6_ molecule in the context of both molecular shape and electrostatic potential.

For C_2_H_4_, two less strong C–H···N hydrogen bonds and two very weak C–H···N electrostatic interactions were observed (Fig. 3b,e and Supplementary Table 4). The strongest one involves one methylene group and one amino group at the narrowest channel neck (C6-H61···N8), while the secondary one involves another methylene group and an uncoordinated triazolate nitrogen atom (C7-H71···N2A), which are also approximately centro-symmetric about the molecular centre. These two C–H···N hydrogen bonds are similar in geometry with the first and third strongest ones for C_2_H_6_. However, their H···N separations (2.54–2.65 Å) are obviously longer, albeit C_2_H_4_ is more polar (Supplementary Table 4). The *cis*-configuration of the two electronegative amino groups and two electropositive methylene groups of the narrow channel neck is crucial for the very different host–guest interactions. Obviously, the molecular geometry of C_2_H_4_ prevents the two hydrogen atoms of a methylene group to form two strong hydrogen bonds with the narrow channel neck like H_2_O and C_2_H_6_. Furthermore, there is significant steric hindrance and electrostatic repulsion between the two C–H moieties of the two methylene groups from the host channel neck and the guest C_2_H_4_ (C3···C6=3.88 Å, Supplementary Fig. 8), which pushes the guest away from the best position for forming a strong C–H···N hydrogen bond with the *p*-position amino group. Conversely, the methylene group of the host channel neck fits well with the threefold symmetric methyl group of C_2_H_6_ (Fig. 3a). For the less strong C–H···N hydrogen bonds and other weak electrostatic attractive interactions, C_2_H_6_ also fits much better with the locations of the electronegative nitrogen atoms, as compared with those for C_2_H_4_ (Fig. 3 and Supplementary Table 4). These observations indicate that the proper locations of both the electronegative nitrogen atoms and the electropositive methylene groups play critical roles in distinguishing C_2_H_6_ and C_2_H_4_ with large adsorption enthalpy difference.

In the simulated host–guest structure for CO_2_, the guest carbon atom locates exactly at the centre of host channel neck, forming short contacts with two free amino groups simultaneously (N···C=2.91 Å), while two oxygen atoms of CO_2_ interact with two methylene groups, respectively, through weak C–H···O hydrogen bonds (C···O=3.33, H···N=2.45 Å, ∠C–H···N=135°) (Fig. 3c,f, Supplementary Table 4). Although these host–guest interactions seem relatively strong, the channel neck diameter (measured by the separation of the *p*-position amino and methylene groups, N8···C3 3.60 Å) significantly expanded from the guest-free state (3.13 Å), while it changes little after loading C_2_H_6_ (3.18 Å) and C_2_H_4_ (3.31 Å), indicating that there is significant steric hindrance and repulsive effect between the CO_2_ molecule and the host framework, and the very short C···N separation is actually the result enforced by the contraction action of the channel neck (Supplementary Fig. 9). It should be noted that all carbon atoms of C_2_H_6_ and C_2_H_4_ reside on one side of the quadrangular channel neck, resulting in much smaller steric hindrance effects compared with CO_2_ (Supplementary Fig. 10).

To confirm the simulation results and directly visualize the host–guest interactions, we carried out SCXRD analyses for MAF-49 loaded with trace amounts of C_2_H_6_, C_2_H_4_ and CO_2_ (denoted as MAF-49·C_2_H_6_, MAF-49·C_2_H_4_ and MAF-49·CO_2_, respectively, see Supplementary Table 2 and Supplementary Data 3,4,5). Compared with the unit-cell volume of guest-free MAF-49, those of MAF-49·C_2_H_6_ and MAF-49·C_2_H_4_ showed minor shrinkage (<0.2%), while that of MAF-49·CO_2_ showed relatively large expansion (1.4%). Further, the N8···C3 separation order of MAF-49, MAF-49·C_2_H_6_, MAF-49·C_2_H_4_ and MAF-49·CO_2_ is consistent with that predicted by computational simulations (Supplementary Fig. 10). In all host–guest crystal structures, the residue electron density peaks can be unambiguously found inside the narrow host channel neck (Fig. 3g–i). Furthermore, in the final crystal structures, all guest molecules locate very similar or identical with those predicted by computational simulations (Supplementary Fig. 10).

### Mixed gas separation

To investigate the practical separation performance of MAF-49, breakthrough experiments were carried out at 313 K and 1 bar. To evaluate and compare the performances of the materials unambiguously, identical column and flow rate were used, and the parameters of each column were optimized (all columns have similar voidage, Supplementary Table 5). Besides, we used the specific injection amount (mmol g^–1^) of the mixed gas as the abscissa, meaning that the breakthrough time (s) was not only divided by the adsorbent weight (g) but also multiplied by the flow rate of the injected mixed gas (mmol s^–1^)^40^.

**Figure 4:**
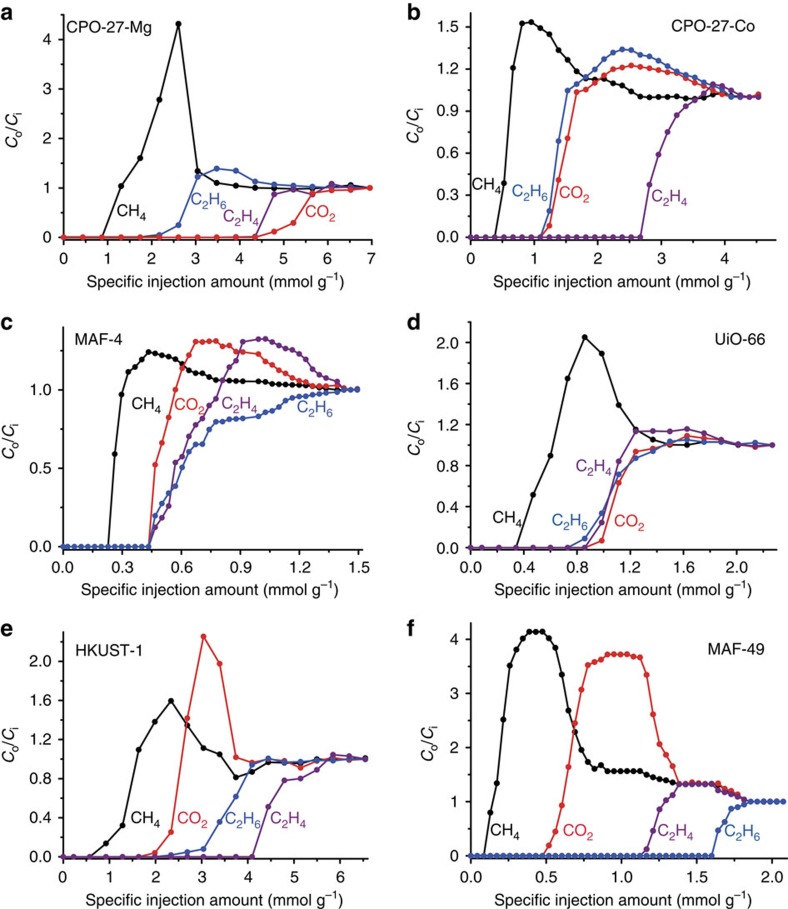
Four-component gas mixture separation. Breakthrough curves of CH_4_/CO_2_/C_2_H_4_/C_2_H_6_ mixture (1:1:1:1 (vol)) for (**a**) CPO-27-Mg, (**b**) CPO-27-Co, (**c**) MAF-4, (**d**) UiO-66, (**e**) HKUST-1 and (**f**) MAF-49 measured at 313 K and 1 bar. Lines are drawn to guide eyes. *C*_i_ and *C*_o_ are the concentrations of each gas at the inlet and outlet, respectively.

To compare the gas adsorption and separation properties of MAF-49 with other protopytical MOFs, breakthrough experiments using an equimolar C_2_H_6_/C_2_H_4_/CO_2_/CH_4_ mixture injection were carried out (Fig. 4 and Supplementary Figs 11 and 12). For MAF-49, a clean and sharp separation of all four gases was observed, while other MOFs showed much poor separation performances and complicated effluent sequences dependent on their pore surface structures. With transition-metal OMSs, [Cu_3_(btc)_2_] (HKUST-1, H_3_btc=benzene-1,3,5-tricarboxylic acid) and [Co_2_(dobdc)] (MOF-74-Co/CPO-27-Co, H_4_dobdc=2,5-dihydroxyl-1,4-benzenedicarboxylic acid) showed binding strength orders C_2_H_4_>C_2_H_6_>CO_2_. Because the main-group-metal OMS tends to form strong interaction with the oxygen atom of CO_2_, [Mg_2_(dobdc)] (MOF-74-Mg/CPO-27-Mg) showed a binding strength order CO_2_>C_2_H_4_>C_2_H_6_. Without pore surface active site, [Zr_6_O_4_(OH)_4_(bdc)_12_] (UiO-66, H_2_bdc=1,4-benzenediarboxylic acid) and [Zn(mim)_2_] (MAF-4 or ZIF-8, Hmim=2-methylimidazole) can barely distinguish the three heavier gases. Nevertheless, the low-polarity adsorbent MAF-4 exhibits slightly better performance compared with UiO-66, and exhibits a separation order similar with that of MAF-49. As expected from the analyses of adsorption isotherms, MAF-49 can also clearly separate two-component C_2_H_4_/C_2_H_6_, C_2_H_6_/CO_2_ and C_2_H_6_/CH_4_ mixtures (Supplementary Fig. 13). It should be noted that C_2_H_6_ could not be detected before its breakthrough points, meaning that C_2_H_6_ is efficiently extracted and high-purity C_2_H_4_/CO_2_/CH_4_ can be obtained directly.

**Figure 5:**
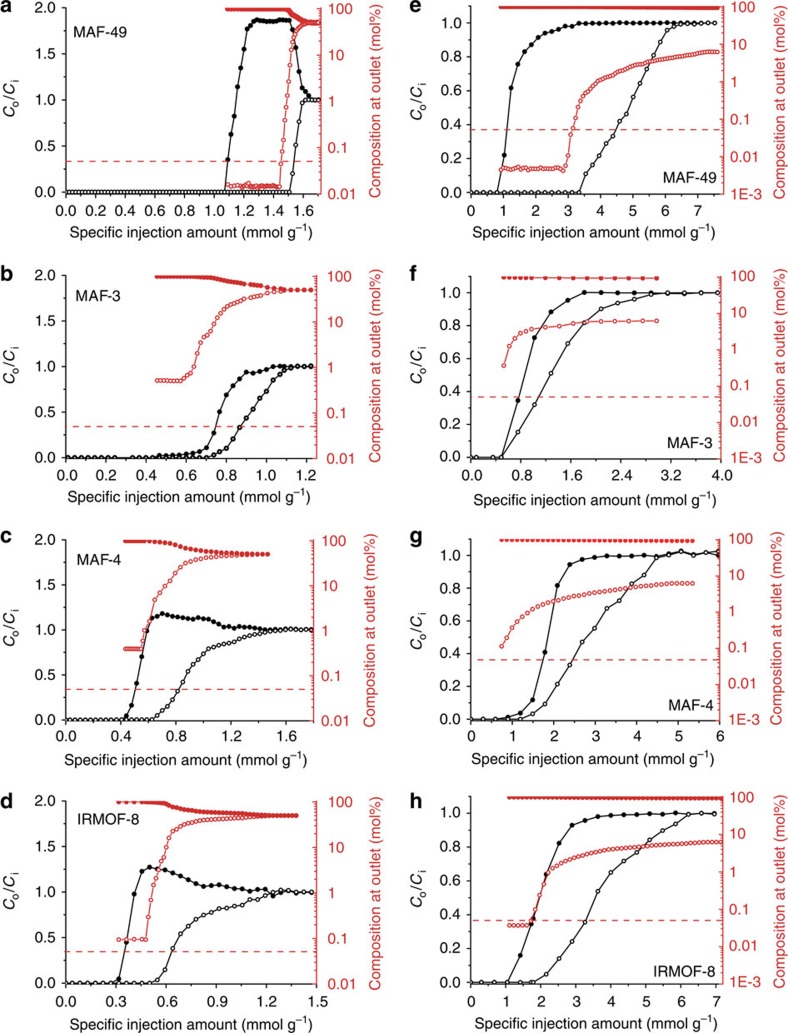
C_2_H_4_/C_2_H_6_ separation performances. C_2_H_4_/C_2_H_6_ (1:1) mixture breakthrough curves of (**a**) MAF-49, (**b**) MAF-3, (**c**) MAF-4 and (**d**) IRMOF-8, and C_2_H_4_/C_2_H_6_ (15:1) mixture breakthrough curves of (**e**) MAF-49, (**f**) MAF-3, (**g**) MAF-4 and (**h**) IRMOF-8 measured at 313 K and 1 bar. Solid symbols: C_2_H_4_, Open symbols: C_2_H_6_. Lines are drawn to guide eyes. *C*_i_ and *C*_o_ are the concentrations of each gas at the inlet and outlet, respectively. Horizontal red dashed lines highlight C_2_H_6_ composition at outlet of 0.05%, that is, C_2_H_4_ purity of 99.95%.

Considering that selective adsorption of C_2_H_6_ over C_2_H_4_ could be beneficial for purification of C_2_H_4_ under fixed-bed adsorption/breakthrough processes, and some hydrophobic MOFs^29^,^30^,^31^,^32^,^33^,^34^,^35^,^36^, such as [Zn(bim)_2_] (MAF-3 or ZIF-7, Hbim=benzimidazole), MAF-4 and [Zn_4_O(ndc)_3_] (IRMOF-8, H_2_ndc=naphthalene-2,6-dicarboxylic acid), were recently reported to exhibit such a property, we compared the C_2_H_4_/C_2_H_6_ adsorption and separation properties of these MOFs with MAF-49 in detail. Single-component C_2_H_6_ adsorption isotherms were measured for MAF-3, MAF-4 and IRMOF-8, which show adsorption enthalpies of 25 kJ mol^−1^, 18 kJ mol^−1^ and 30 kJ mol^−1^, respectively (Fig. 2c and Supplementary Fig. 14), at zero loading, being much lower than that of MAF-49. Although the C_2_H_6_ uptake at 1 bar for MAF-49 (38 cm^3^ g^−1^) is lower than that of the more porous adsorbents IRMOF-8 (91 cm^3^ g^−1^), MAF-4 (48 cm^3^ g^−1^) and MAF-3 (41 cm^3^ g^−1^), its C_2_H_6_ uptake at 0.06 bar (36 cm^3^ g^−1^) is *ca*. 4 times that of IRMOF-8 (9 cm^3^ g^−1^), 19 times that of MAF-4 (1.9 cm^3^ g^−1^) and 45 times that of MAF-3 (0.8 cm^3^ g^−1^) (Fig. 2d). Considering that a purity of 100% is impossible and the C_2_H_6_ concentration before its breakthrough point is lower than the detection limit of the conventionally used thermal conductivity detector, the gas stream at the column outlet was analysed with a mass spectrometer (MS). For a 1:1 C_2_H_4_/C_2_H_6_ mixture injection (Fig. 5a and Supplementary Fig. 15a), the breakthrough points of C_2_H_4_ and C_2_H_6_ for MAF-49 were observed by thermal conductivity detector at 1.09 and 1.44 mmol g^−1^, respectively, during which the C_2_H_6_ concentration was determined as 0.014–0.016% by MS, corresponding to a C_2_H_4_ purity of 99.986–99.984% (Fig. 5a and Supplementary Figs 15a and 16). Under identical conditions, the highest C_2_H_4_ purities achieved by MAF-3, MAF-4 and IRMOF-8 are only 99.5%, 99.6% and 99.9% (C_2_H_6_ concentrations of 0.5%, 0.4% and 0.1%), respectively, reflecting their much lower C_2_H_6_/C_2_H_4_ selectivity compared with MAF-49 (Fig. 5b–d, Supplementary Table 6 and Supplementary Figs 15–19). Nevertheless, such C_2_H_4_ purities are obviously higher than those reported for C_2_H_4_ selective adsorbent materials (99%+)^13^,^17^,^23^,^24^, which exemplify the feasibility of using C_2_H_6_ selective adsorbents for purifying C_2_H_4_, because the desired gas can be continuously purified by passing through the column and directly obtained from the first effluents. Indeed, desorbing the MAF-49 column saturated by 1:1 C_2_H_4_/C_2_H_6_ mixture can give C_2_H_6_ with 99%+ purity with a peak value of only 99.7% (Supplementary Fig. 20). A realistic comparison for the C_2_H_4_ purification performance of different adsorbents, of relevance to industrial operations, can be obtained by comparing the breakthrough amount of C_2_H_4_ (denoted as productivity) with the desired purity in a single breakthrough operation (for the calculation method see Supplementary Methods). For the MAF-49 column, 0.28 mmol g^−1^ or 0.44 mol l^−1^ of C_2_H_4_ with 99.95%+ purity can be recovered from a 1:1 C_2_H_4_/C_2_H_6_ mixture injection. For the MAF-3, MAF-4 and IRMOF-8 columns, their productivities are zero because the C_2_H_4_ effluents are not pure enough. Even for a C_2_H_4_ purity of 99%+, the productivity of the MAF-49 column (0.32 mmol g^−1^ or 0.47 mol l^−1^) is still much higher than the others (the largest value is 0.11 mmol g^−1^ or 0.10 mol l^−1^) (Supplementary Table 6).

Since the C_2_H_6_ concentration in C_2_H_4_/C_2_H_6_ mixtures produced by hydrocarbon cracking is just *ca*. 5–9% (refs 41, 42, 43), breakthrough experiments using a 15:1 C_2_H_4_/C_2_H_6_ mixture injection were carried out. The lowest C_2_H_6_ impurity or highest C_2_H_4_ purities achieved by the MAF-49, MAF-3, MAF-4 and IRMOF-8 columns are decreased to 0.005%, 0.4%, 0.1% and 0.04% or improved to 99.995%, 99.6%, 99.9% and 99.96%, respectively (Fig. 5e–h and Supplementary Figs 21–24). Obviously, using C_2_H_6_ selective adsorbents, the C_2_H_4_ purity can be increased by lengthening the adsorbent bed (increasing adsorbent amount), which is simpler and more convenient than the C_2_H_4_ selective adsorbents^13^,^17^,^25^. For the 15:1 C_2_H_4_/C_2_H_6_ mixture injection and the C_2_H_4_ output purity of 99.95%+, the MAF-49 column gave a C_2_H_4_ productivity of 1.68 mmol g^−1^ or 2.48 mol l^−1^, which is about 30 or 50 times that of IRMOF-8 (0.06 mmol g^−1^ or 0.05 mol l^−1^), in the gravimetric or volumetric point-of-view, respectively (Supplementary Table 7). Note that for C_2_H_4_ purification, the adsorbent volume is more practical than its weight because the fixed-bed equipment does not need to move during operation. For lower C_2_H_4_ purities such as 99.5%+ and 99%+, the C_2_H_4_ productivities of MAF-49 and IRMOF-8 were also increased (Supplementary Tables 6 and 7), because the adsorber needs more time to reach adsorption saturation for the mixture gas containing low-concentration C_2_H_6_. Nevertheless, the C_2_H_4_ productivity of MAF-49 improved more significantly than for IRMOF-8 at all purity standards (Supplementary Tables 6 and 7), because the former material exhibits much higher C_2_H_6_ uptakes at the low pressure region (Fig. 2c). In contrast, the MAF-3 and MAF-4 columns only showed slightly increased C_2_H_4_ purities (did not reach 99.95%+) at a C_2_H_4_/C_2_H_6_ feeding ratio of 15:1 (Fig. 2c), because lengthening the adsorber is not so effective to improve the effluent purity by using adsorbents with weak impurity affinity. For C_2_H_4_ purities of 99.5%+ and 99%+, the C_2_H_4_ productivities of the MAF-3 and MAF-4 columns obtained by using a 15:1 C_2_H_4_/C_2_H_6_ input were unexpectedly lower than for the 1:1 C_2_H_4_/C_2_H_6_ mixture (Supplementary Tables 6 and 7), which can be attributed to the extremely low C_2_H_6_ adsorption ability of the adsorbents at the low pressure region. Also, the partial pressures of C_2_H_4_ and C_2_H_6_ in the 15:1 C_2_H_4_/C_2_H_6_ mixture are not beneficial for utilizing the differential gate-opening effect of MAF-3 (ref. 44).

## Discussion

In summary, we reported a unique adsorbent material showing selective adsorption of C_2_H_6_ over more polar analogous molecules such as C_2_H_4_ and CO_2_, which can be useful for extraction of C_2_H_6_ from natural gases and particularly valuable for direct production of high-purity C_2_H_4_ from C_2_H_4_/C_2_H_6_ mixtures. The key to this C_2_H_6_ selectivity is a combination of multiple hydrogen-bonding acceptors and dipole repulsion groups locating at appropriate positions on the pore surface of a very narrow channel, which not only allows multiple attractive interactions for C_2_H_6_ but also enforces C_2_H_4_ to adopt a position that can only form fewer and weaker attractive interactions. In short, this work provides not only a new MOF with exceptionally high C_2_H_4_ separation/purification performances, but also a new molecular design strategy for developing next-generation adsorbents.

## Methods

### Materials and general methods

Reagents and solvents were commercially available and were used without further purification, H_2_batz (ref. 45), MAF-3 (ref. 30), MAF-4 (ref. 46), IRMOF-8 (ref. 47), HKUST-1 (ref. 48), CPO-27-Mg (ref. 49), CPO-27-Co (ref. 49) and UiO-66 (ref. 50) were synthesized according to the literature methods. Elemental analyses (C, H, N) were performed with a Vario EL elemental analyzer. Thermogravimetry analysis was performed under N_2_ with temperature increased with 5 °C min^–1^ using a TA-Q50 system. Powder XRD patterns were collected on a Bruker D8 Advance diffractometer (Cu Kα) at room temperature.

### Synthesis of [Zn(batz)]·0.5H_2_O (MAF-49·H_2_O)

A mixture of Zn(OH)_2_ (0.100 g, 1.0 mmol), H_2_batz (0.180 g, 1.0 mmol), aqueous ammonia (25%, 4 ml) and water (4 ml) was stirred for 15 min in air, then transferred and sealed in a 15 ml Teflon reactor, which was heated in an oven at 160 °C for 72 h. The oven was cooled to room temperature at a rate of 5 °C h^−1^. The resulting colourless block crystals were filtered, washed and dried in air (yield *ca*. 86%). Anal. Calcd (%) for C_5_H_7_N_8_O_0.5_Zn: C, 23.77; H, 2.79; N, 44.36. Found: C, 23.97; H, 2.82; N, 44.13. Guest-free MAF-49 was obtained by heating the as-synthesized sample under high vacuum at 150 °C for 12 h.

### Single-crystal X-ray crystallography

Diffraction intensities were collected on a Pilatus XtaLAB P300DS diffractometer with graphite-monochromated Mo Kα radiation. Absorption corrections were applied by using the multi-scan programme REQAB. The structures were solved by the direct method and refined with the full-matrix least-squares technique using the SHELXTL programme package. It should be noted that, because the molecular centres of the very short C_2_H_6_ and C_2_H_4_ molecules do not locate at the centre of the two-fold symmetric host channel neck as predicted by computational simulations, their molecular geometries have to be restricted during refinement of the crystal structures. Also, the positions of their hydrogen atoms were added according to the computational simulation result. Because of disorder and low occupancies of the gas molecules, anisotropic thermal parameters were only applied to all non-hydrogen atoms of the host framework. Crystal data for the compounds were summarized in Supplementary Table 2. Electron density maps were generated using the output from standard SHELXL refinements in a number of ways using WinGX and VESTA 3.0.8.

### Gas sorption measurement

The sorption isotherms were measured with an automatic volumetric adsorption apparatus (BELSORP-max). The as-synthesized sample (about 200−300 mg) was placed in the sample tube and dried for 12 h at 320 °C to remove the remnant solvent molecules prior to measurement. CO_2_ (99.999%), C_2_H_4_ (99.95%), CH_4_ (99.999%) and C_2_H_6_ (99.99%) were used for all adsorption isotherm and breakthrough experiments (Supplementary Fig. 25). The temperatures were controlled by a water bath (298, 307 and 316 K).

## Additional information

**Accession codes:** The X-ray crystallographic coordinates for structures reported in this Article have been deposited at the Cambridge Crystallographic Data Centre (CCDC), under deposition number CCDC 1421354-1421358. These data can be obtained free of charge from the Cambridge Crystallographic Data Centre via www.ccdc.cam.ac.uk/data_request/cif.

**How to cite this article:** Liao, P.-Q. *et al*. Efficient purification of ethene by an ethane-trapping metal-organic framework. *Nat. Commun.* 6:8697 doi: 10.1038/ncomms9697 (2015).

## Supplementary Material

Supplementary Information Supplementary Figures 1-25, Supplementary Tables 1-7, Supplementary Methods and Supplementary References

Supplementary Data 1 X-ray single-crystal structures of as-synthesized MAF-49 in CIF format

Supplementary Data 2 X-ray single-crystal structures of guest-free MAF-49 in CIF format

Supplementary Data 3 X-ray single-crystal structures of C2H6 included MAF-49 in CIF format

Supplementary Data 4 X-ray single-crystal structures of C2H4 included MAF-49 in CIF format

Supplementary Data 5 X-ray single-crystal structures of CO2 included MAF-49 in CIF format

## References

[b1] Matar S. & Hatch L. F. Chemistry of Petrochemical Processes 2nd edn Gulf Publishing Company (2000).

[b2] Baker R. W. Future directions of membrane gas separation technology. Ind. Eng. Chem. Res. 41, 1393–1411 (2002).

[b3] Horike S. . Dense coordination network capable of selective CO_2_ capture from C1 and C2 hydrocarbons. J. Am. Chem. Soc. 134, 9852–9855 (2012). 22667323 10.1021/ja302043u

[b4] Zhou J. . Removal of C_2_H_4_ from a CO_2_ stream by adsorption: a study in combination of ab initio calculation and experimental approach. Energy Fuels 20, 778–782 (2006).

[b5] Liao P.-Q., Zhu A.-X., Zhang W.-X., Zhang J.-P. & Chen X.-M. Self-catalysed aerobic oxidization of organic linker in porous crystal for on-demand regulation of sorption behaviours. Nat. Commun. 6, 6350 (2015). 25702689 10.1038/ncomms7350

[b6] Worrell E., Phylipsen D., Einstein D. & Martin N. Energy Use and Energy Intensity of the U.S. Chemical Industry. Report No. LBNL-44314 (Energy Analysis Department, Environmental Energy Technologies Division, Ernest Orlando Lawrence Berkeley National Laboratory, University of California, Berkeley, California 9472, USA, 2000).

[b7] Ren T., Patel M. & Blok K. Olefins from conventional and heavy feedstocks: energy use in steam cracking and alternative processes. Energy 31, 425–451 (2006).

[b8] Safarik D. J. & Eldridge R. B. Olefin/paraffin separations by reactive absorption: a review. Ind. Eng. Chem. Res. 37, 2571–2581 (1998).

[b9] Yang R. T. Adsorbents: Fundamentals and Applications 191–230John Wiley & Sons, Inc. (2003).

[b10] Tanaka K., Taguchi A., Hao J., Kita H. & Okamoto K. Permeation and separation properties of polyimide membranes to olefins and paraffins. J. Membr. Sci. 121, 197–207 (1996).

[b11] Bux H., Chmelik C., Krishna R. & Caro J. Ethene/ethane separation by the MOF membrane ZIF-8: molecular correlation of permeation, adsorption, diffusion. J. Membr. Sci. 369, 284–289 (2011).

[b12] Uchida S. . Selective sorption of olefins by halogen-substituted macrocation-polyoxometalate porous ionic crystals. Chem. Mater. 24, 325–330 (2012).

[b13] Li B. . Introduction of *π*-complexation into porous aromatic framework for highly selective adsorption of ethylene over ethane. J. Am. Chem. Soc. 136, 8654–8660 (2014). 24901372 10.1021/ja502119z

[b14] Uchida S., Kawamoto R., Tagami H., Nakagawa Y. & Mizuno N. Highly selective sorption of small unsaturated hydrocarbons by nonporous flexible framework with silver ion. J. Am. Chem. Soc. 130, 12370–12376 (2008). 18722424 10.1021/ja801453c

[b15] Denysenko D., Grzywa M., Jelic J., Reuter K. & Volkmer D. Scorpionate-type coordination in MFU-4l metal–organic frameworks: small-molecule binding and activation upon the thermally activated formation of open metal sites. Angew. Chem. Int. Ed. 53, 5832–5836 (2014). 10.1002/anie.201310004 24846505

[b16] Aguado S., Bergeret G., Daniel C. & Farrusseng D. Absolute molecular sieve separation of ethylene/ethane mixtures with silver zeolite A. J. Am. Chem. Soc. 134, 14635–14637 (2012). 22913514 10.1021/ja305663k

[b17] Bloch E. D. . Hydrocarbon separations in a metal-organic framework with open iron(ii) coordination sites. Science 335, 1606–1610 (2012). 22461607 10.1126/science.1217544

[b18] Herm Z. R., Bloch E. D. & Long J. R. Hydrocarbon separations in metal–organic frameworks. Chem. Mater. 26, 323–338 (2014).

[b19] Geier S. J. . Selective adsorption of ethylene over ethane and propylene over propane in the metal-organic frameworks M_2_(dobdc) (M=Mg, Mn, Fe, Co, Ni, Zn). Chem. Sci. 4, 2054–2061 (2013).

[b20] Zhang Y. . Highly selective adsorption of ethylene over ethane in a MOF featuring the combination of open metal site and *π*-complexation. Chem. Commun. 51, 2714–2717 (2015). 10.1039/c4cc09774b 25575193

[b21] Bao Z. . Adsorption of ethane, ethylene, propane, and propylene on a magnesium-based metal–organic framework. Langmuir 27, 13554–13562 (2011). 21942644 10.1021/la2030473

[b22] Yang S. . Supramolecular binding and separation of hydrocarbons within a functionalized porous metal–organic framework. Nat. Chem. 7, 121–129 (2015). 25615665 10.1038/nchem.2114

[b23] He Y., Krishna R. & Chen B. Metal-organic frameworks with potential for energy-efficient adsorptive separation of light hydrocarbons. Energy Environ. Sci. 5, 9107–9120 (2012).

[b24] Rege S. U., Padin J. & Yang R. T. Olefin/paraffin separations by adsorption: π-Complexation *vs*. kinetic separation. AIChE J. 44, 799–809 (1998).

[b25] Silva F. A. D. & Rodrigues A. E. Propylene/propane separation by vacuum swing adsorption using 13X zeolite. AIChE J. 47, 341–357 (2001).

[b26] Brookhart M., Findlater M., Guironnet D. & Lyons T. W. Synthesis of para-xylene and toluene. US Patent 13/875,610 (2013).

[b27] Vogler D. E. & Sigrist M. W. Near-infrared laser based cavity ringdown spectroscopy for applications in petrochemical industry. Appl. Phys. B 85, 349–354 (2006).

[b28] Meyers R. & Meyers R. A. Handbook of Petrochemicals Production Processes McGraw-Hill Prof Med/Tech (2005).

[b29] Böhme U. . Ethene/ethane and propene/propane separation via the olefin and paraffin selective metal–organic framework adsorbents CPO-27 and ZIF-8. Langmuir 29, 8592–8600 (2013). 23802617 10.1021/la401471g

[b30] Gücüyener C., van den Bergh J., Gascon J. & Kapteijn F. Ethane/ethene separation turned on its head: selective ethane adsorption on the metal−organic Framework ZIF-7 through a gate-opening mechanism. J. Am. Chem. Soc. 132, 17704–17706 (2010). 21114318 10.1021/ja1089765

[b31] Pires J., Pinto M. L. & Saini V. K. Ethane selective IRMOF-8 and its significance in ethane–ethylene separation by adsorption. ACS Appl. Mater. Interfaces 6, 12093–12099 (2014). 25010787 10.1021/am502686g

[b32] Cai J. . A doubly interpenetrated metal–organic framework with open metal sites and suitable pore sizes for highly selective separation of small hydrocarbons at room temperature. Cryst. Growth Des. 13, 2094–2097 (2013).

[b33] He Y. . A microporous metal–organic framework for highly selective separation of acetylene, ethylene, and ethane from methane at room temperature. Chem. Eur. J. 18, 613–619 (2012). 22162259 10.1002/chem.201102734

[b34] He Y. . A robust doubly interpenetrated metal-organic framework constructed from a novel aromatic tricarboxylate for highly selective separation of small hydrocarbons. Chem. Commun. 48, 6493–6495 (2012). 10.1039/c2cc31792c 22622325

[b35] Das M. C. . A Zn_4_O-containing doubly interpenetrated porous metal-organic framework for photocatalytic decomposition of methyl orange. Chem. Commun. 47, 11715–11717 (2011). 10.1039/c1cc12802g 21952516

[b36] He Y. . A microporous lanthanide-tricarboxylate framework with the potential for purification of natural gas. Chem. Commun. 48, 10856–10858 (2012). 10.1039/c2cc35729a 23023243

[b37] Li J.-R., Sculley J. & Zhou H.-C. Metal–organic frameworks for separations. Chem. Rev. 112, 869–932 (2011). 21978134 10.1021/cr200190s

[b38] Zhang J.-P., Zhang Y.-B., Lin J.-B. & Chen X.-M. Metal azolate frameworks: from crystal engineering to functional materials. Chem. Rev. 112, 1001–1033 (2012). 21939178 10.1021/cr200139g

[b39] Myers A. L. & Prausnitz J. M. Thermodynamics of mixed-gas adsorption. AIChE. J. 11, 121–126 (1965).

[b40] Liao P.-Q. . Monodentate hydroxide as a super strong yet reversible active site for CO_2_ capture from high-humidity flue gas. Energy Environ. Sci. 8, 1011–1016 (2015).

[b41] Sedighi M., Keyvanloo K. & Darian T. J. Olefin production from heavy liquid hydrocarbon thermal cracking: kinetics and product distribution. Iran. J. Chem. Chem. Eng. 29, 135–147 (2010).

[b42] Marquevich M., Coll R. & Montané D. Steam reforming of sunflower oil for hydrogen production. Ind. Eng. Chem. Res. 39, 2140–2147 (2000).

[b43] Guo J., Wu X., Jing S., Zhang Q. & Zheng D. Vapor-liquid equilibrium of ethylene+mesitylene system and process simulation for ethylene recovery. Chin. J. Chem. Eng. 19, 543–548 (2011).

[b44] van den Bergh J. . Understanding the anomalous alkane selectivity of ZIF-7 in the separation of light alkane/alkene mixtures. Chem. Eur. J. 17, 8832–8840 (2011). 21755546 10.1002/chem.201100958

[b45] Dippold A. A., Feller M. & Klapoetke T. M. 5,5'-dinitrimino-3,3'-methylene-1H-1,2,4-bistriazole - a metal free primary explosive combining excellent thermal stability and high performance. Cent. Eur. J. Energ. Mater. 8, 261–278 (2011).

[b46] Huang X.-C., Lin Y.-Y., Zhang J.-P. & Chen X.-M. Ligand-directed strategy for zeolite-type metal–organic frameworks: Zinc(II) imidazolates with unusual zeolitic topologies. Angew. Chem. Int. Ed. 45, 1557–1559 (2006). 10.1002/anie.200503778 16440383

[b47] Feldblyum J. I., Wong-Foy A. G. & Matzger A. J. Non-interpenetrated IRMOF-8: synthesis, activation, and gas sorption. Chem. Commun. 48, 9828–9830 (2012). 10.1039/c2cc34689c 22930156

[b48] Chui S.S.-Y., Lo S.M.-F., Charmant J. P. H., Orpen A. G. & Williams I. D. A chemically functionalizable nanoporous material [Cu_3_(TMA)_2_(H_2_O)_3_]_n_. Science 283, 1148–1150 (1999). 10024237 10.1126/science.283.5405.1148

[b49] Caskey S. R., Wong-Foy A. G. & Matzger A. J. Dramatic tuning of carbon dioxide uptake via metal substitution in a coordination polymer with cylindrical pores. J. Am. Chem. Soc. 130, 10870–10871 (2008). 18661979 10.1021/ja8036096

[b50] Cavka J. H. . A new zirconium inorganic building brick forming metal organic frameworks with exceptional stability. J. Am. Chem. Soc. 130, 13850–13851 (2008). 18817383 10.1021/ja8057953

