# Supplementary material for: Efficient purification of ethene by an ethane-trapping metal-organic framework
Source: Nat Commun. 2015 Oct 29;6:8697. doi: 10.1038/ncomms9697 (PMC4846320; doi:10.1038/ncomms9697)
Supplement: Supplementary Information — Supplementary Figures 1-25, Supplementary Tables 1-7, Supplementary Methods and Supplementary References [file ncomms9697-s1.pdf]

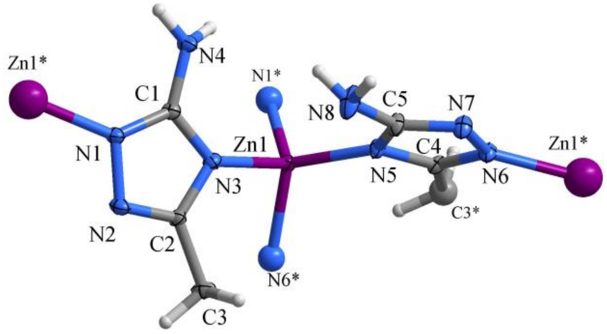

Supplementary Figure 1. The local coordination environment in MAF-49·H_2O (probability drawn at 30%). * represents symmetrically generated atoms.

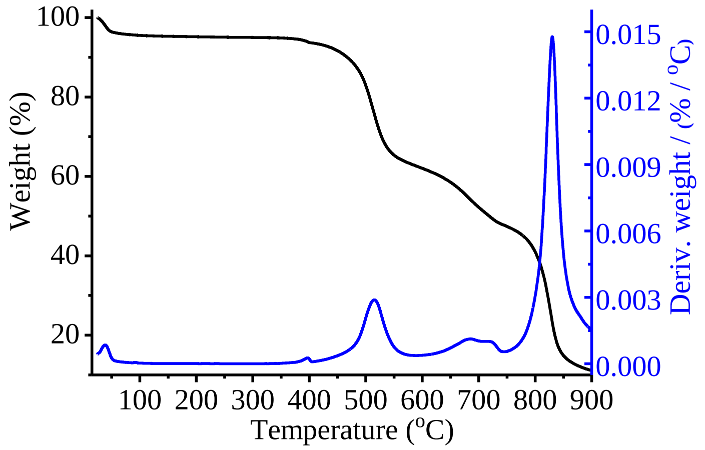

Supplementary Figure 2. TG curve of MAF-49·H_2O.

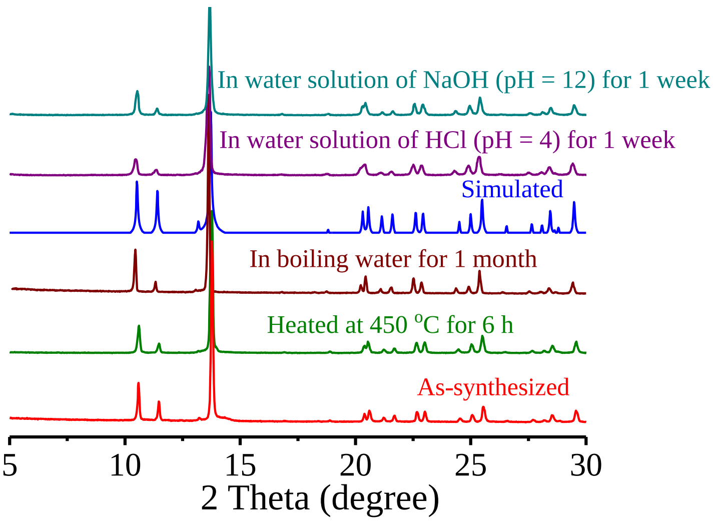

Supplementary Figure 3. PXRD patterns of MAF-49 after heating/adsorption/desorption treatments.

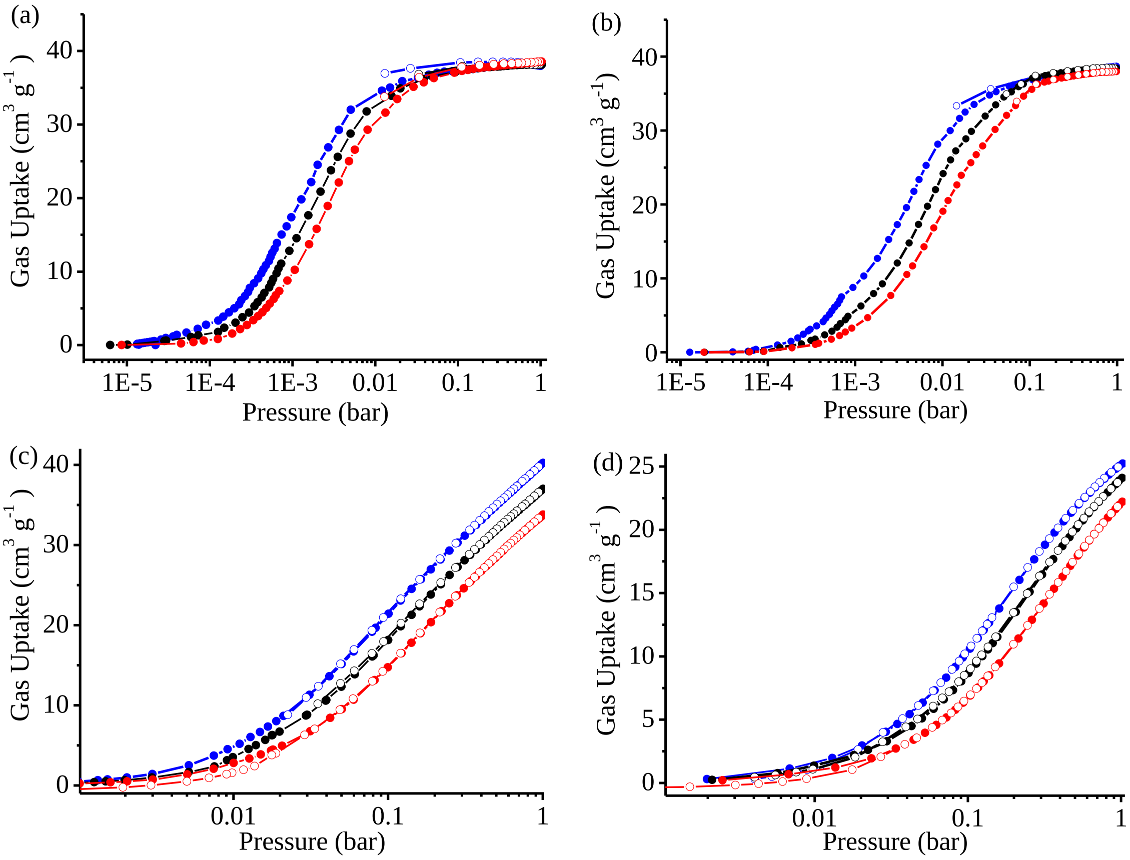

Supplementary Figure 4. (a) C_2H_6, (b) C_2H_4, (c) CO_2 and (d) CH_4 adsorption (solid) and desorption (open) isotherms of MAF-49 measured at 298 (blue), 307 (black) and 316 (red) K.

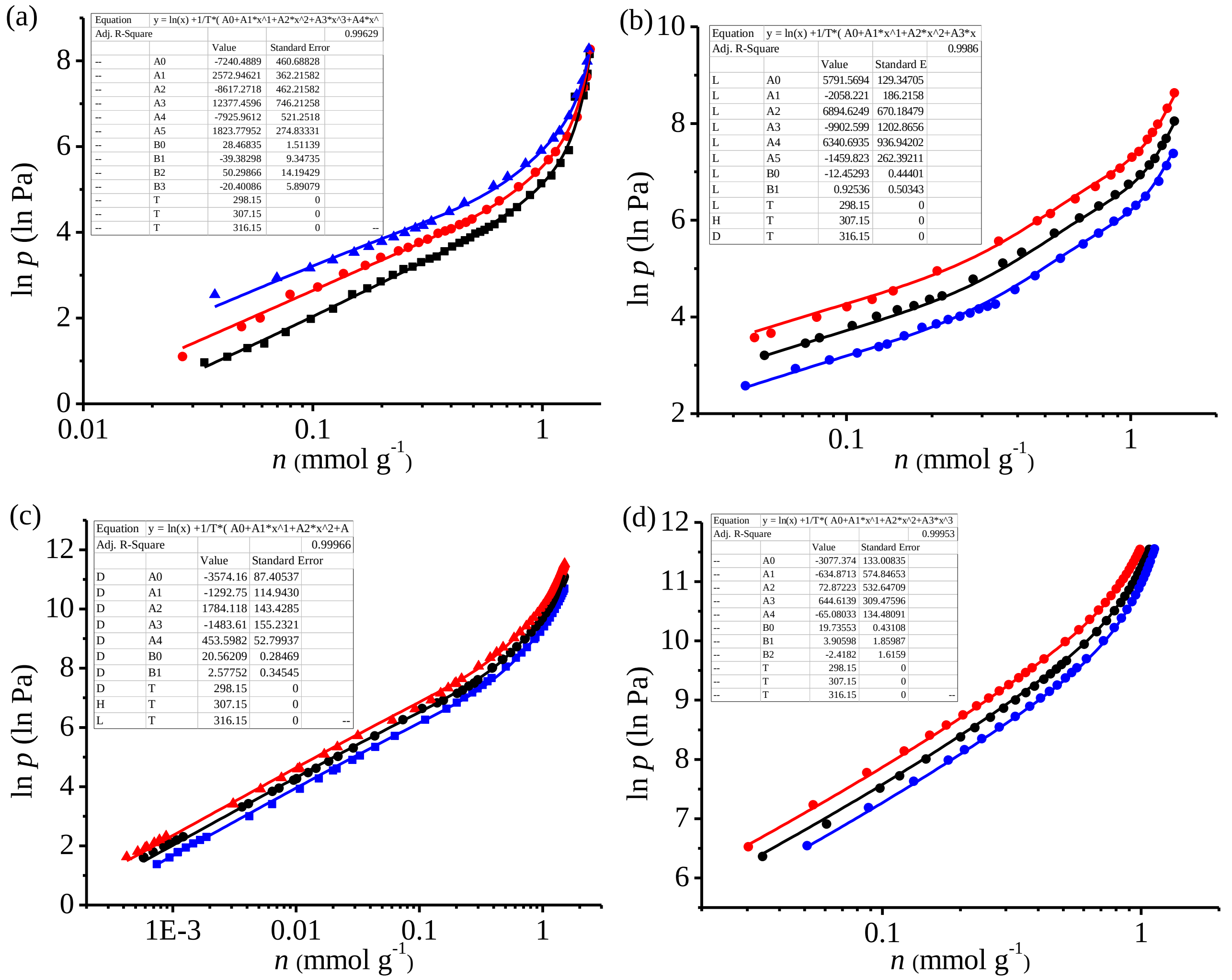

Supplementary Figure 5. Virial fitting (lines) of the (a) C_2H_6, (b) C_2H_4, (c) CO_2 and (d) CH_4 adsorption isotherms (points) of MAF-49 measured at 298 (blue), 307 (black) and 316 (red) K.

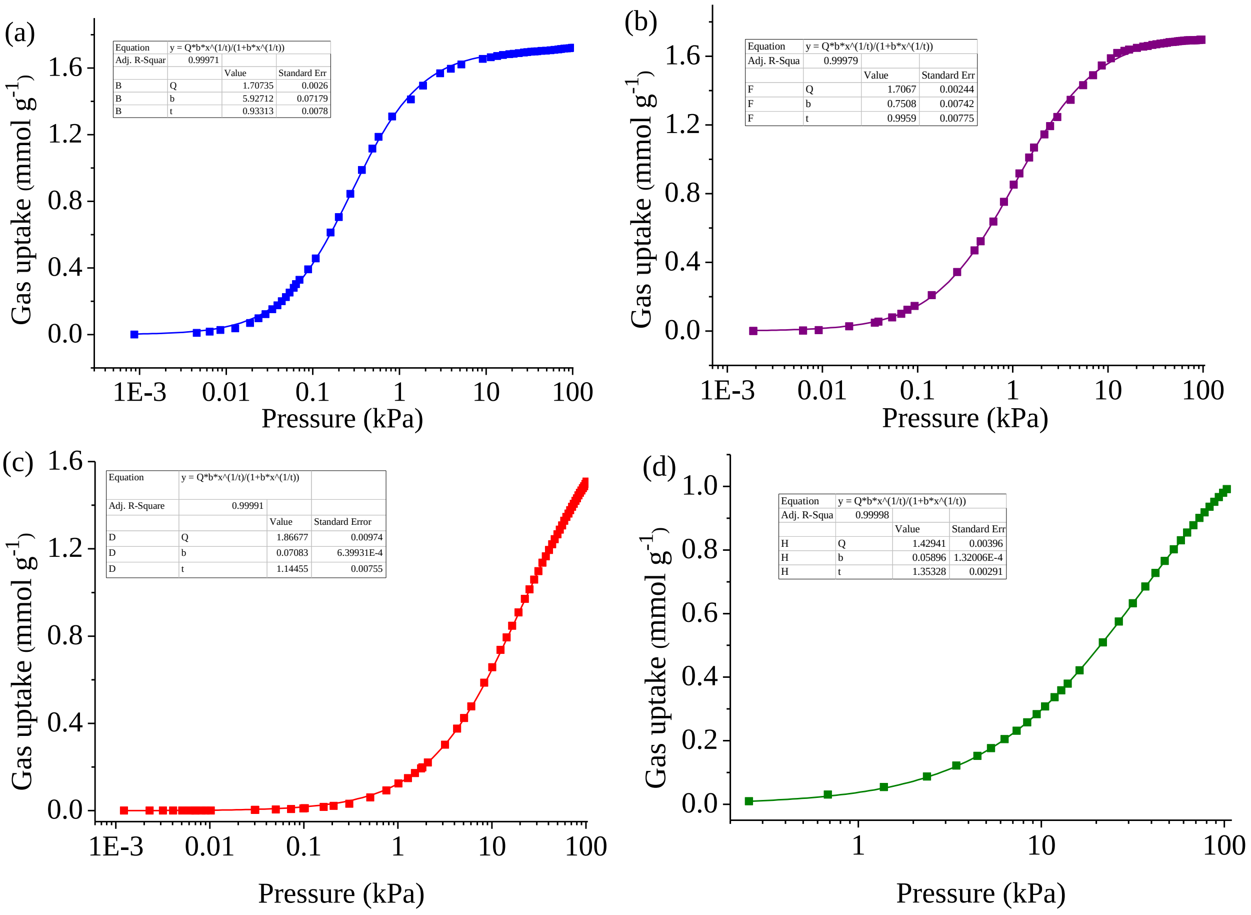

Supplementary Figure 6. Langmuir−Freundlich fitting (lines) of the (a) C_2H_6, (b) C_2H_4, (c) CO_2 and (d) CH_4 adsorption isotherms (points) of MAF-49 measured at 316 K.

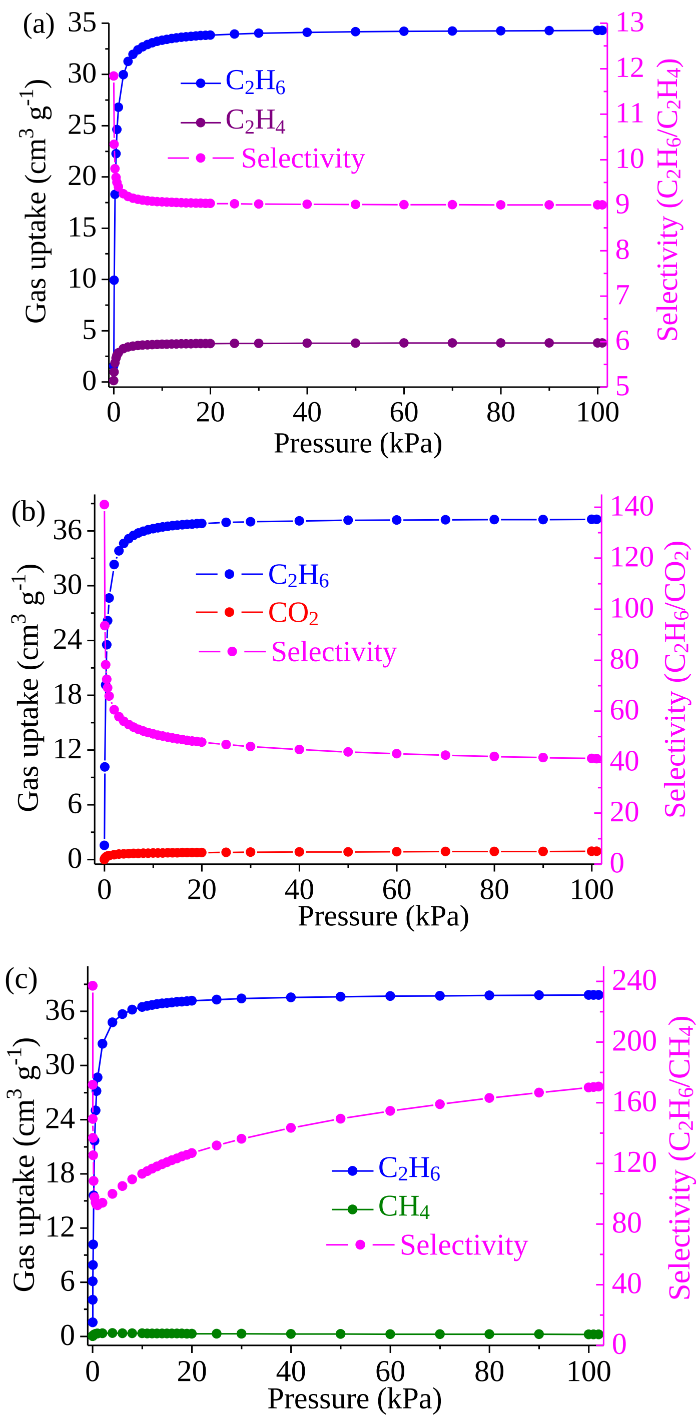

Supplementary Figure 7. Mixed isotherms and selectivity in MAF-49 predicted by IAST for equimolar mixtures of (a) C_2H_6/C_2H_4, (b) C_2H_6/CO_2 and (c) C_2H_6/CH_4 at 316 K.

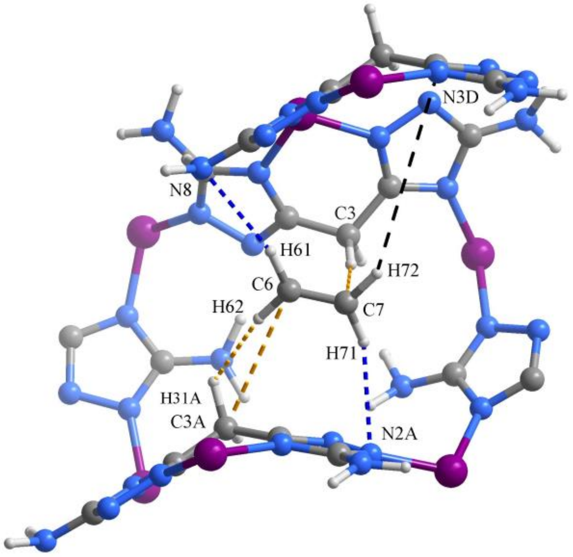

Supplementary Figure 8. The steric hindrance and electrostatic repulsion between the two C-H moieties of the two methylene groups from the host channel neck and the guest C_2H_4 (C3A···C6 = 3.88 Å, H31A···H62 = 2.10 Å) in MAF-49·C_2H_4.

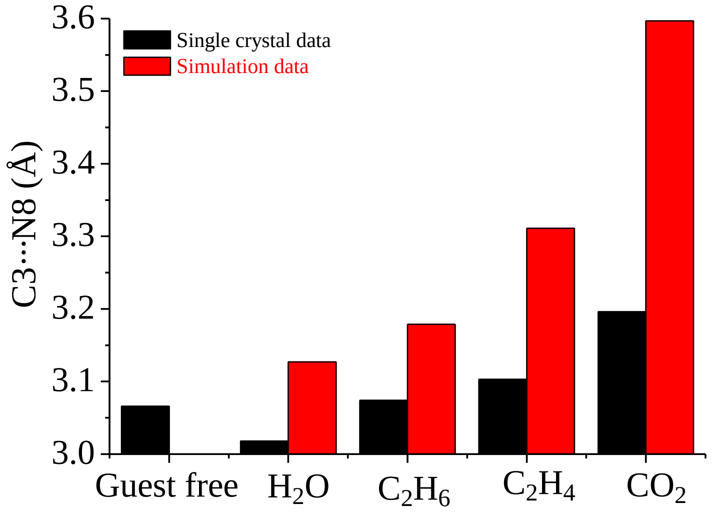

Supplementary Figure 9. Comparison of the channel neck sizes (defined by The N8···C3 separation as shown in Figure 3) of MAF-49, MAF-49·C_2H_6, MAF-49·C_2H_4, MAF-49·CO_2 obtained by computational simulations and single-crystal X-ray diffraction.

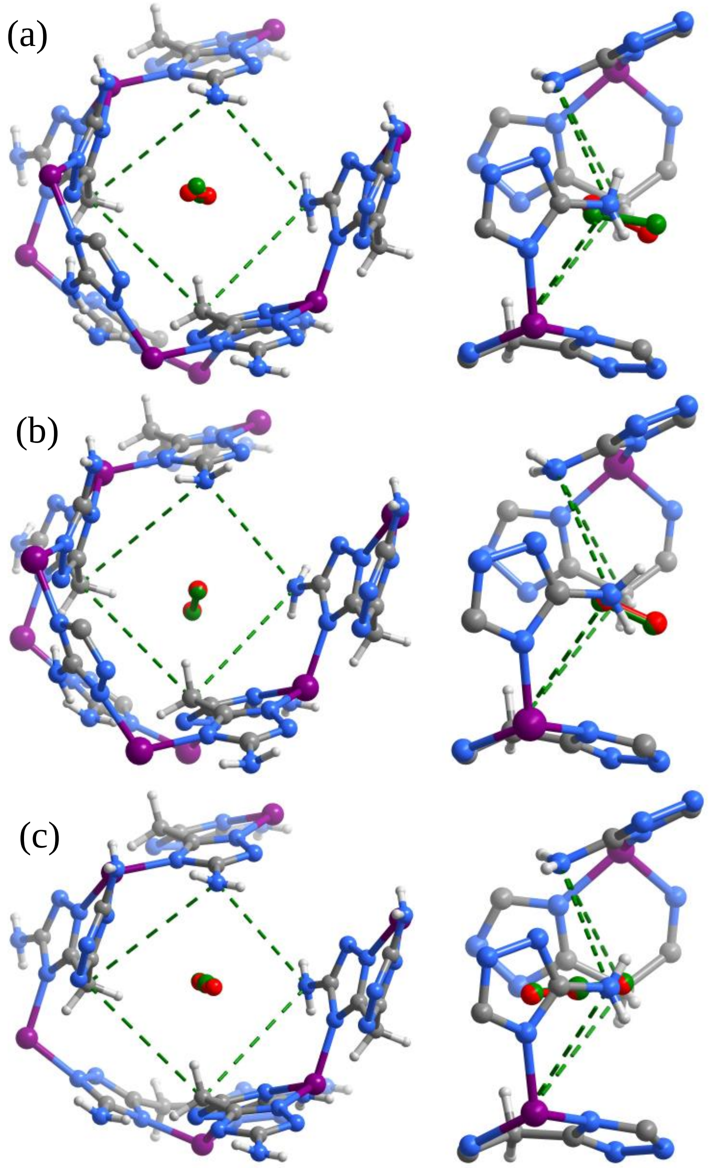

Supplementary Figure 10. Comparison of the preferential (a) C_2H_6, (b) C_2H_4 and (c) CO_2 adsorption sites (Zn purple, C dark gray, H light gray, N blue, hydrogen atoms are omitted for clarity) observed by single-crystal X-ray diffraction (red) and PDFT calculation (green). Left and Right, the structures are projected along the channel neck and perpendicular to the channel neck, respectively.

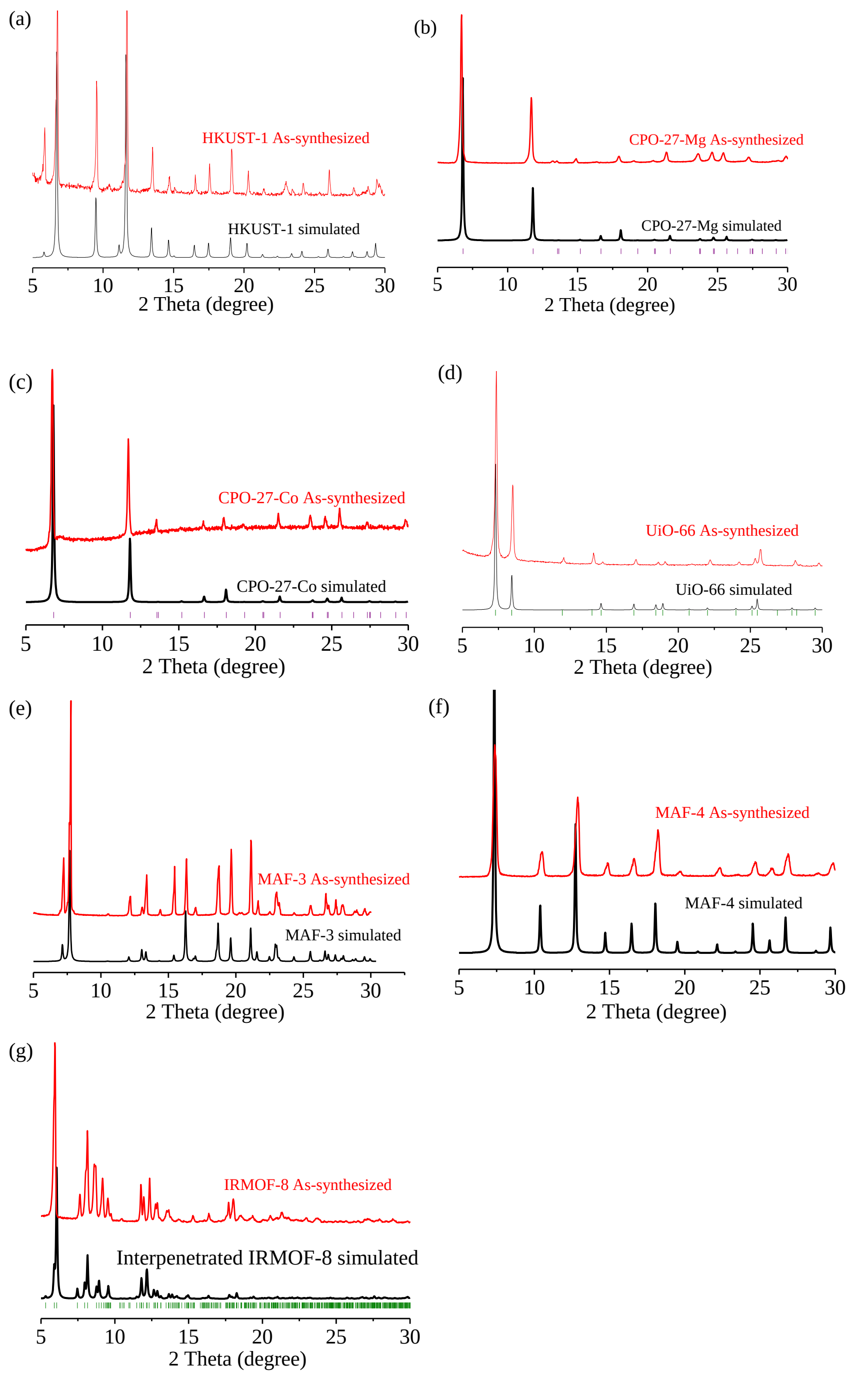

Supplementary Figure 11. PXRD patterns of (a) HKUST-1, (b) MOF-74-Mg, (c) MOF-74-Co, (d) UiO-66, (e) MAF-3, (f) MAF-4 and (g) IRMOF-8 samples used in this work. Note that the IRMOF-8 sample adopt an interpenetrated structure, which has been commonly obtained in the literature and used for gas (such as H_2[1] and C_2H_6/C_2H_4[2, 3]) adsorption studies.

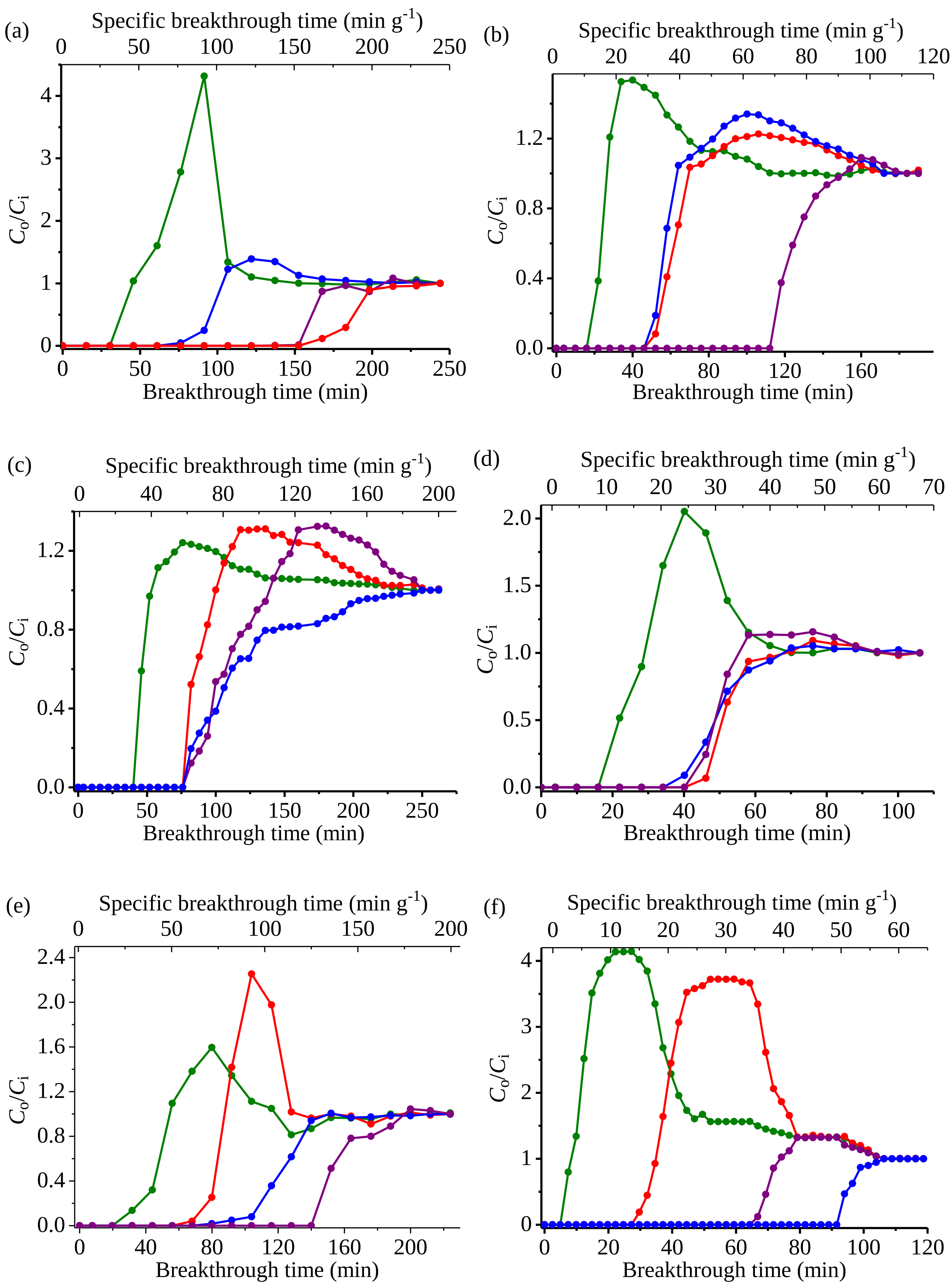

Supplementary Figure 12. Breakthrough curves of Figure 4 expressed using time (min) and specific breakthrough time (min g^{-1}) as abscissa.

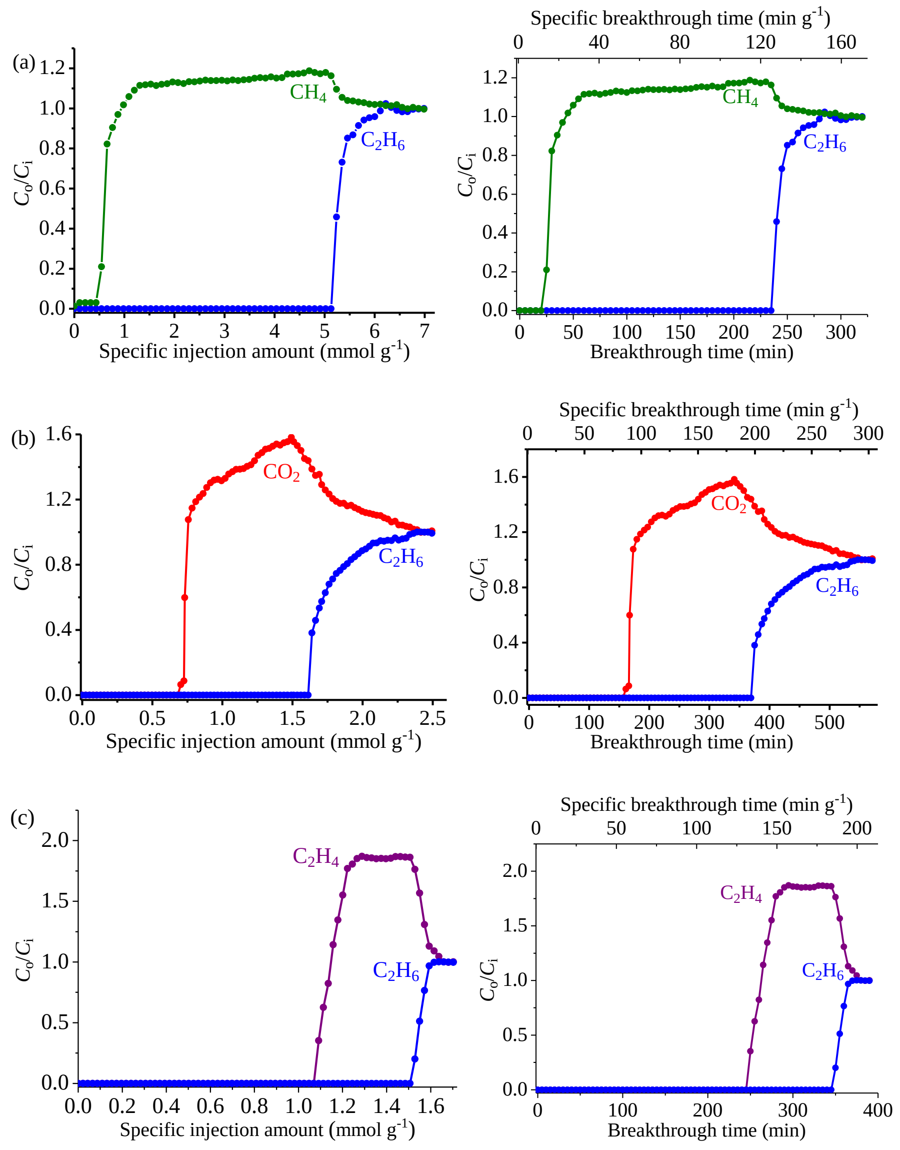

Supplementary Figure 13. Breakthrough curves of (a) 4:1 CH_4/C_2H_6 mixture, (b) 1:1 CO_2/C_2H_6 mixture, and (c) 1:1 C_2H_4/C_2H_6 mixture for MAF-49. Left, specific injection amount (mmol g^{-1}) as abscissa. Right, specific breakthrough time (min g^{-1}) and breakthrough time (min) as abscissa. Lines are drawn to guide eyes. C_i and C_o are the concentrations of each gas at the inlet and outlet, respectively.

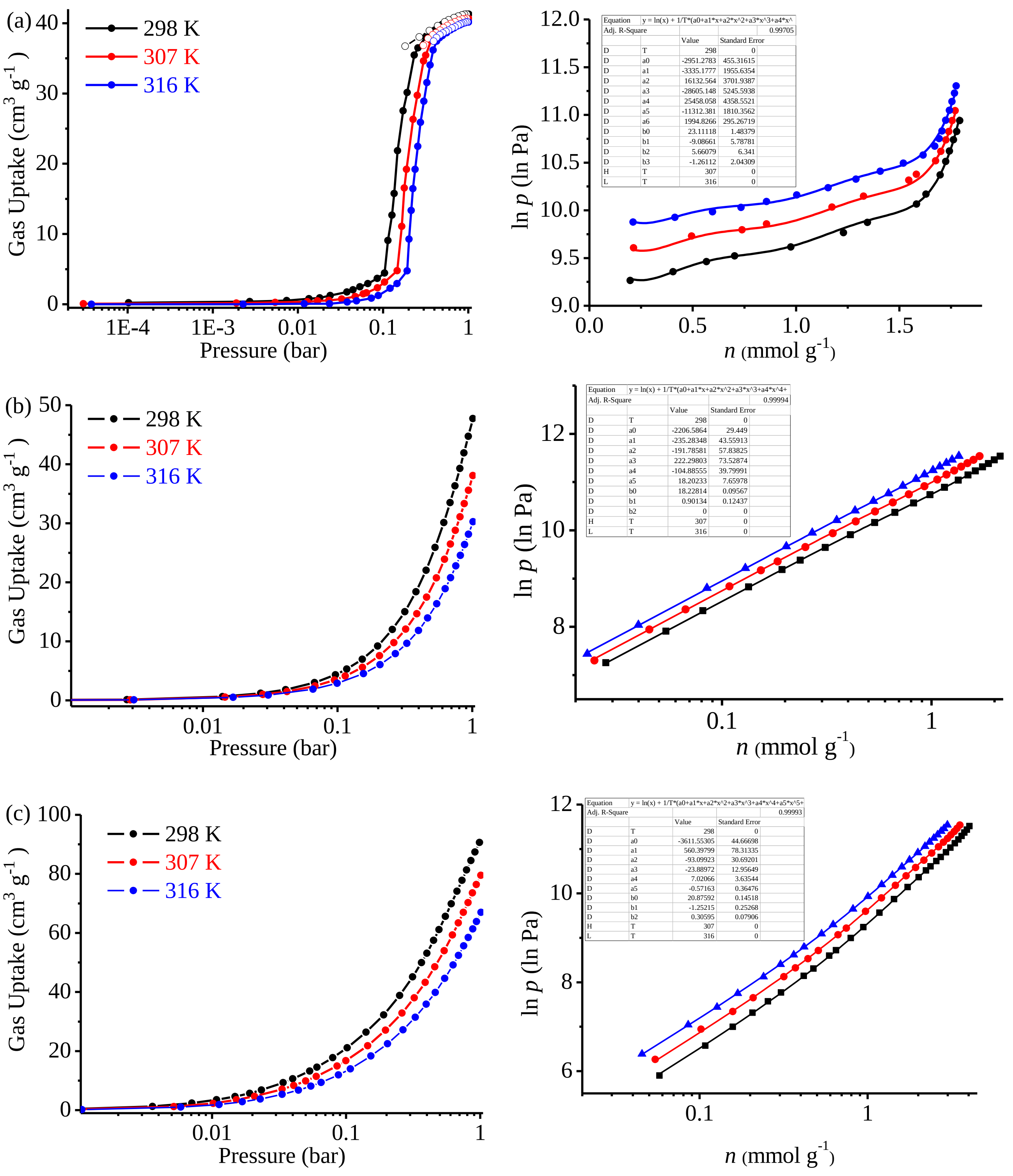

Supplementary Figure 14. The C_2H_6 adsorption isotherms (left, lines are drawn to guide eyes) and Virial fitting (right, lines are fitting results) of (a) MAF-3, (b) MAF-4 and (c) IRMOF-8.

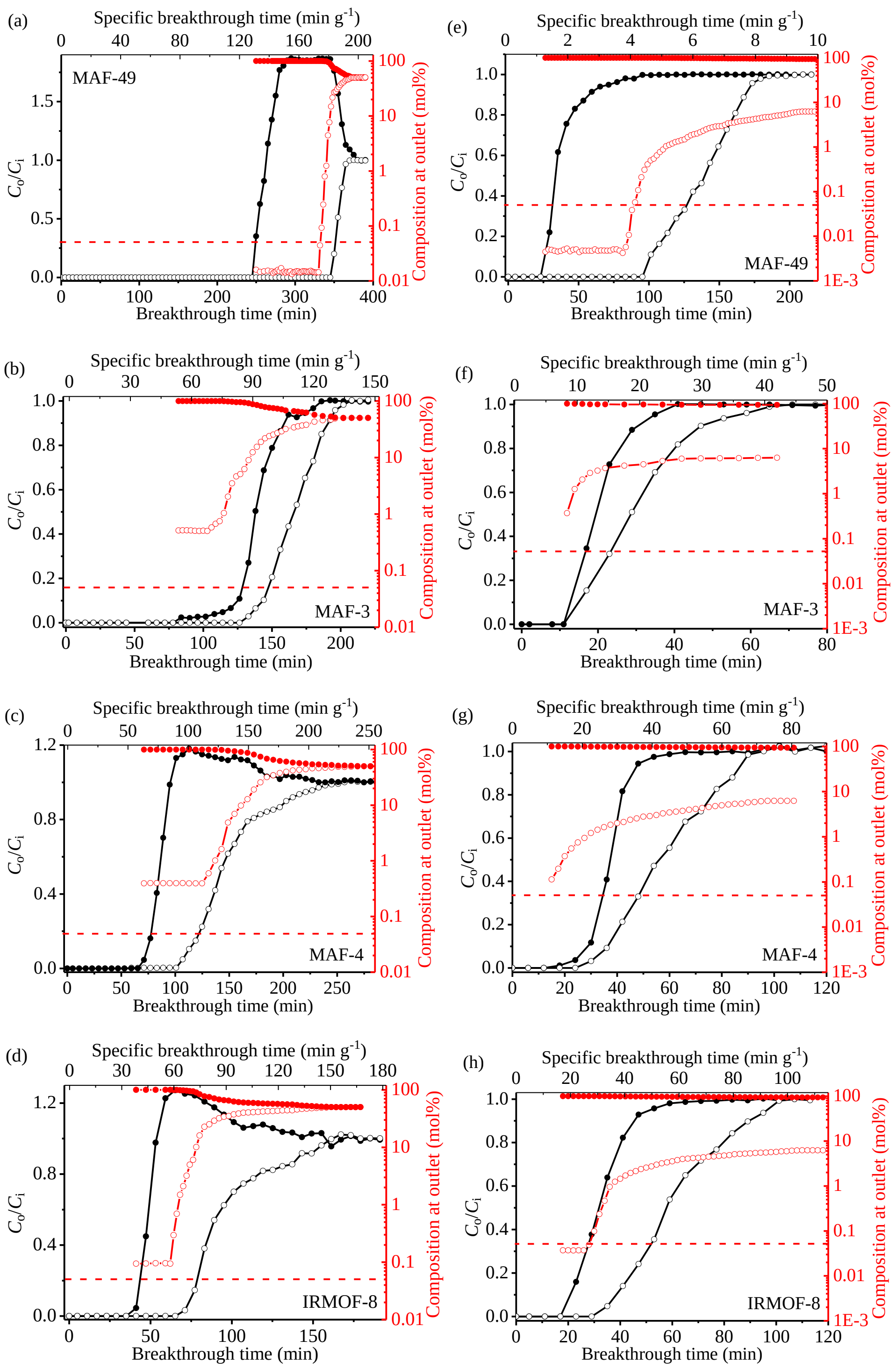

Supplementary Figure 15. Breakthrough curves of Figure 5 expressed using breakthrough time (min) and specific breakthrough time (min g^{-1}) as abscissa.

Area Percent Report

Data Path : H:\LiaoPQ-GC-MS-20150526-R2\
Data File : MAF-49-1-1-C2H4-C2H6-MS-017.D
Acq On : 31 Mar 2015 20:58
Operator :
Sample :
Misc :
ALS Vial : 1 Sample Multiplier: 1

Integration Parameters: rteint.p
Integrator: RTE
Smoothing : ON Filtering: 5
Sampling : 1 Min Area: 3 % of largest Peak
Start Thrs: 0.2 Max Peaks: 100
Stop Thrs : 0 Peak Location: TOP

If leading or trailing edge < 100 prefer < Baseline drop else tangent >
Peak separation: 5

Method : E:\data\Jennifer\Flow throught system\GC methods\5PAHs_3Pesticides_liquid injection_splitless.M
Title :

Signal : EIC Ion 28.00 (27.70 to 28.70): MAF-49-1-1-C2H4-C2H6-MS-017.D\data.ms

peak #	R.T. min	first scan	max scan	last scan	PK TY	peak height	corr. area	corr. % max.	% of total
1	0.919	306	321	394	rM 4	2402897	64363059	100.00%	100.000%

Sum of corrected areas: 64363059

Signal : EIC Ion 30.00 (29.70 to 30.70): MAF-49-1-1-C2H4-C2H6-MS-017.D\data.ms

peak #	R.T. min	first scan	max scan	last scan	PK TY	peak height	corr. area	corr. % max.	% of total
1	0.912	307	318	363	rM 4	3536	9848	100.00%	100.000%

Sum of corrected areas: 9848

5PAHs_3Pest..._splitless.M Thu May 28 09:54:48 2015

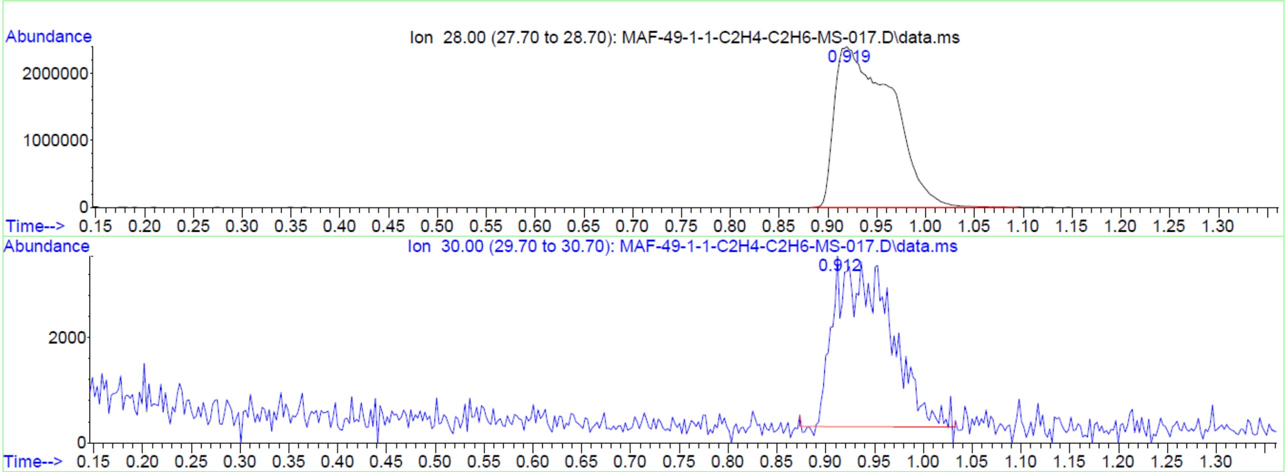

Supplementary Figure 16. GC-MS spectra of the outlet gas (top: C_2H_4, bottom C_2H_6) showing C_2H_4 purity of 99.98% given by MAF-49 for a single breakthrough operation using 1:1 C_2H_4/C_2H_6 as input.

Area Percent Report

Data Path : H:\LiaoPQ-GC-MS-20150526-R2\
Data File : MAF-3-1-1-C2H4-C2H6-010.D
Acq On : 3 Apr 2015 8:20
Operator :
Sample :
Misc :
ALS Vial : 1 Sample Multiplier: 1

Integration Parameters: rteint.p
Integrator: RTE
Smoothing : ON Filtering: 5
Sampling : 1 Min Area: 3 % of largest Peak
Start Thrs: 0.2 Max Peaks: 100
Stop Thrs : 0 Peak Location: TOP

If leading or trailing edge < 100 prefer < Baseline drop else tangent >
Peak separation: 5

Method : E:\data\Jennifer\Flow throught system\GC methods\5PAHs_3Pesticides_liquid injection_splitless.M
Title :

Signal : EIC Ion 28.00 (27.70 to 28.70): MAF-3-1-1-C2H4-C2H6-010.D\data.ms

peak #	R.T. min	first scan	max scan	last scan	PK TY	peak height	corr. area	corr. % max.	% of total
1	0.973	336	351	407	rBV3	7522564	136112261	100.00%	100.000%

Sum of corrected areas: 136112261

Signal : EIC Ion 30.00 (29.70 to 30.70): MAF-3-1-1-C2H4-C2H6-010.D\data.ms

peak #	R.T. min	first scan	max scan	last scan	PK TY	peak height	corr. area	corr. % max.	% of total
1	0.973	340	351	376	rVB3	47344	497102	100.00%	100.000%

Sum of corrected areas: 497102

5PAHs_3Pest..._splitless.M Thu May 28 10:01:32 2015

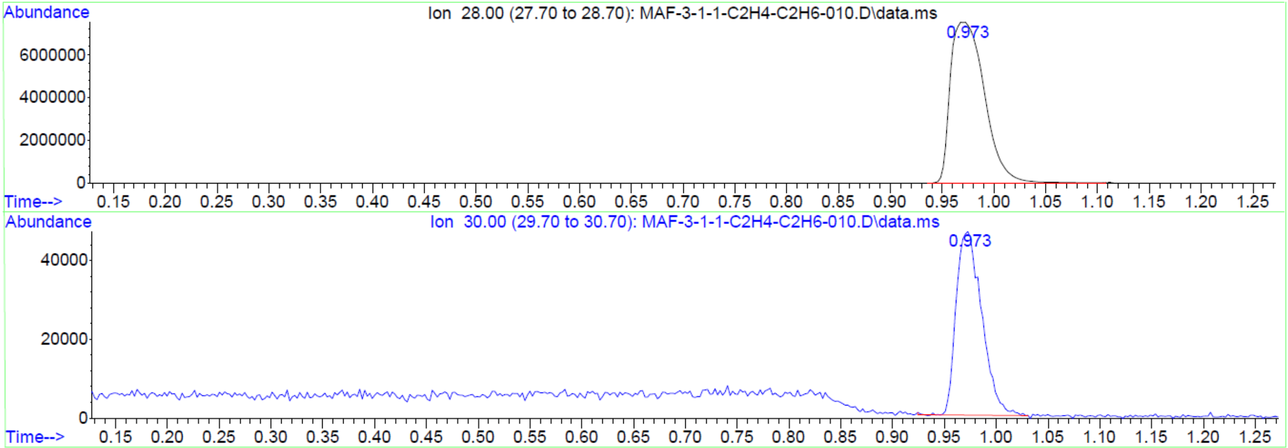

Supplementary Figure 17. GC-MS spectra of the outlet gas (top: C_2H_4, bottom C_2H_6) with C_2H_4 purity of 99.5% given by MAF-3 for a single breakthrough operation using 1:1 C_2H_4/C_2H_6 as input.

Area Percent Report

Data Path : H:\LiaoPQ-GC-MS-20150526-R2\
Data File : MAF-4-1-1-C2H4-C2H6-001.D
Acq On : 1 Apr 2015 17:34
Operator :
Sample :
Misc :
ALS Vial : 1 Sample Multiplier: 1

Integration Parameters: rteint.p
Integrator: RTE
Smoothing : ON Filtering: 5
Sampling : 1 Min Area: 3 % of largest Peak
Start Thrs: 0.2 Max Peaks: 100
Stop Thrs : 0 Peak Location: TOP

If leading or trailing edge < 100 prefer < Baseline drop else tangent >
Peak separation: 5

Method : E:\data\Jennifer\Flow throught system\GC methods\5PAHs_3Pesticides_liquid injection_splitless.M
Title :

Signal : EIC Ion 28.00 (27.70 to 28.70): MAF-4-1-1-C2H4-C2H6-001.D\data.ms

peak #	R.T. min	first scan	max scan	last scan	PK TY	peak height	corr. area	corr. % max.	% of total
1	0.965	329	347	389	rM 5	3243520	44270812	100.00%	100.000%

Sum of corrected areas: 44270812

Signal : EIC Ion 30.00 (29.70 to 30.70): MAF-4-1-1-C2H4-C2H6-001.D\data.ms

peak #	R.T. min	first scan	max scan	last scan	PK TY	peak height	corr. area	corr. % max.	% of total
1	0.963	335	346	366	rM 5	68997	142241	100.00%	100.000%

Sum of corrected areas: 142241

5PAHs_3Pest..._splitless.M Thu May 28 09:58:21 2015

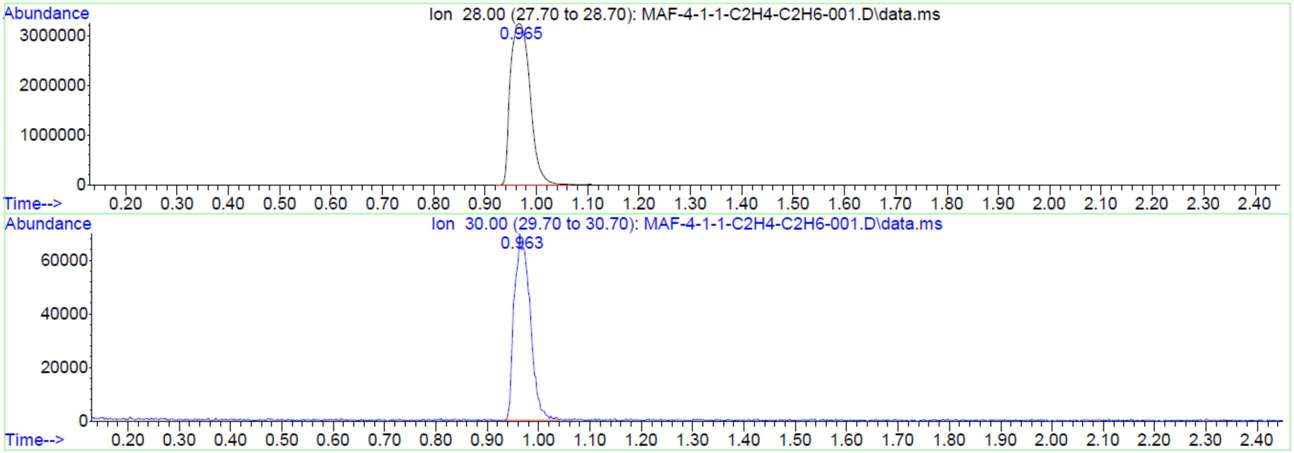

Supplementary Figure 18. GC-MS spectra of the outlet gas (top: C_2H_4, bottom C_2H_6) with C_2H_4 purity of 99.6% given by MAF-4 for a single breakthrough operation using 1:1 C_2H_4/C_2H_6 as input.

Area Percent Report

Data Path : H:\LiaoPQ-GC-MS-20150526-R2\
Data File : IRMOF-8-1-1-C2H4-C2H6-MS-05.D
Acq On : 31 Mar 2015 23:07
Operator :
Sample :
Misc :
ALS Vial : 1 Sample Multiplier: 1

Integration Parameters: rteint.p
Integrator: RTE
Smoothing : ON Filtering: 5
Sampling : 1 Min Area: 3 % of largest Peak
Start Thrs: 0.2 Max Peaks: 100
Stop Thrs : 0 Peak Location: TOP

If leading or trailing edge < 100 prefer < Baseline drop else tangent >
Peak separation: 5

Method : E:\data\Jennifer\Flow throught system\GC methods\5PAHs_3Pesticides_liquid injection_splitless.M
Title :

Signal : EIC Ion 28.00 (27.70 to 28.70): IRMOF-8-1-1-C2H4-C2H6-MS-05.D\data.ms

peak #	R.T. min	first scan	max scan	last scan	PK TY	peak height	corr. area	corr. % max.	% of total
1	0.884	288	308	423	rM 4	6957820	549710224	100.00%	100.000%

Sum of corrected areas: 549710224

Signal : EIC Ion 30.00 (29.70 to 30.70): IRMOF-8-1-1-C2H4-C2H6-MS-05.D\data.ms

peak #	R.T. min	first scan	max scan	last scan	PK TY	peak height	corr. area	corr. % max.	% of total
1	0.892	297	311	370	rM 4	10959	439160	100.00%	100.000%

Sum of corrected areas: 439160

5PAHs_3Pest..._splitless.M Thu May 28 10:00:05 2015

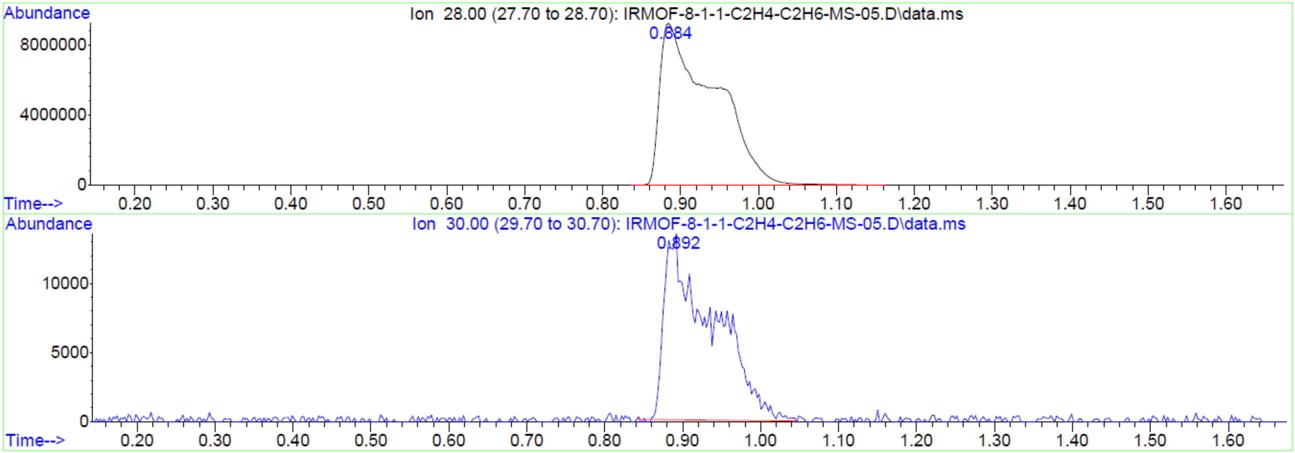

Supplementary Figure 19. GC-MS spectra of the outlet gas (top: C_2H_4, bottom C_2H_6) with C_2H_4 purity of 99.9% given by IRMOF-8 for a single breakthrough operation using 1:1 C_2H_4/C_2H_6 as input.

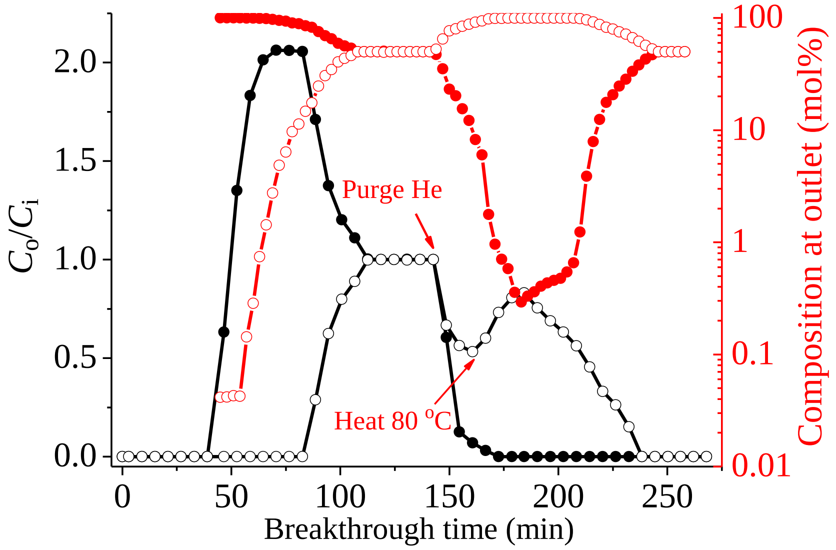

Supplementary Figure 20. Breakthrough curves of a complete adsorption-desorption cycle using 1:1 C_2H_4/C_2H_6 mixture for MAF-49 (1.346 g sample was used, corresponding to an apparent density of 0.811 g cm^{-3} a column voidage of 0.452; The mixed gas and He flow rates are both 0.6 cm^3 min^{-1}). Solid symbols: C_2H_4, Open symbols: C_2H_6. Lines are drawn to guide eyes. C_i and C_o are the concentrations of each gas at the inlet and outlet, respectively.

Area Percent Report

Data Path : H:\LiaoPQ-GC-MS-20150526-R2\
Data File : MAF-49-15-1-C2H4-C2H6-011.D
Acq On : 4 Apr 2015 21:11
Operator :
Sample :
Misc :
ALS Vial : 1 Sample Multiplier: 1

Integration Parameters: rteint.p
Integrator: RTE
Smoothing : ON Filtering: 5
Sampling : 1 Min Area: 3 % of largest Peak
Start Thrs: 0.2 Max Peaks: 100
Stop Thrs : 0 Peak Location: TOP

If leading or trailing edge < 100 prefer < Baseline drop else tangent >
Peak separation: 5

Method : E:\data\Jennifer\Flow throught system\GC methods\5PAHs_3Pesticides_liquid injection_splitless.M
Title :

Signal : EIC Ion 28.00 (27.70 to 28.70): MAF-49-15-1-C2H4-C2H6-011.D\data.ms

peak #	R.T. min	first scan	max scan	last scan	PK TY	peak height	corr. area	corr. % max.	% of total
1	0.971	319	343	384	rM 2	4851775	135146886	100.00%	100.000%

Sum of corrected areas: 135146886

Signal : EIC Ion 30.00 (29.70 to 30.70): MAF-49-15-1-C2H4-C2H6-011.D\data.ms

peak #	R.T. min	first scan	max scan	last scan	PK TY	peak height	corr. area	corr. % max.	% of total
1	0.971	320	343	365	rM 2	2723	5575	100.00%	100.000%

Sum of corrected areas: 5575

5PAHs_3Pest..._splitless.M Thu May 28 10:10:31 2015

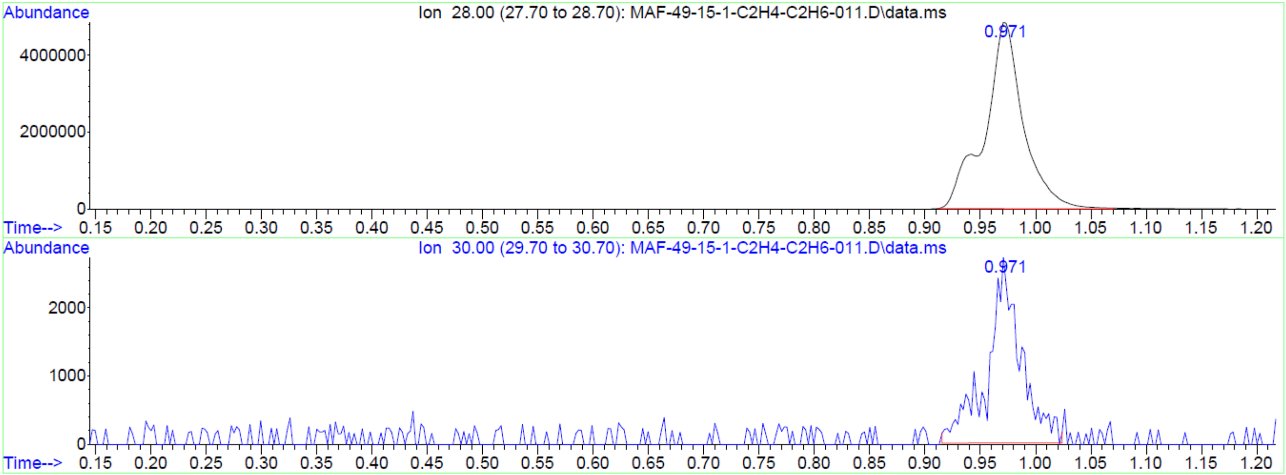

Supplementary Figure 21. GC-MS spectra of the outlet gas (top: C_2H_4, bottom C_2H_6) with C_2H_4 purity of 99.995% given by MAF-49 for a single breakthrough operation using 15:1 C_2H_4/C_2H_6 as input.

Area Percent Report

Data Path : H:\LiaoPQ-GC-MS-20150526-R2\
Data File : MAF-3-15-1-C2H4-C2H6-013.D
Acq On : 5 Apr 2015 9:02
Operator :
Sample :
Misc :
ALS Vial : 1 Sample Multiplier: 1

Integration Parameters: rteint.p
Integrator: RTE
Smoothing : ON Filtering: 5
Sampling : 1 Min Area: 3 % of largest Peak
Start Thrs: 0.2 Max Peaks: 100
Stop Thrs : 0 Peak Location: TOP

If leading or trailing edge < 100 prefer < Baseline drop else tangent >
Peak separation: 5

Method : E:\data\Jennifer\Flow throught system\GC methods\5PAHs_3Pesticides_liquid injection_splitless.M
Title :

Signal : EIC Ion 28.00 (27.70 to 28.70): MAF-3-15-1-C2H4-C2H6-013.D\data.ms

peak #	R.T. min	first scan	max scan	last scan	PK TY	peak height	corr. area	corr. % max.	% of total
1	0.989	339	351	400	rBV2	291584	2571186	100.00%	100.000%

Sum of corrected areas: 2571186

Signal : EIC Ion 30.00 (29.70 to 30.70): MAF-3-15-1-C2H4-C2H6-013.D\data.ms

peak #	R.T. min	first scan	max scan	last scan	PK TY	peak height	corr. area	corr. % max.	% of total
1	0.989	343	351	374	rM 2	5191	8602	100.00%	100.000%

Sum of corrected areas: 8602

5PAHs_3Pest..._splitless.M Thu May 28 10:04:43 2015

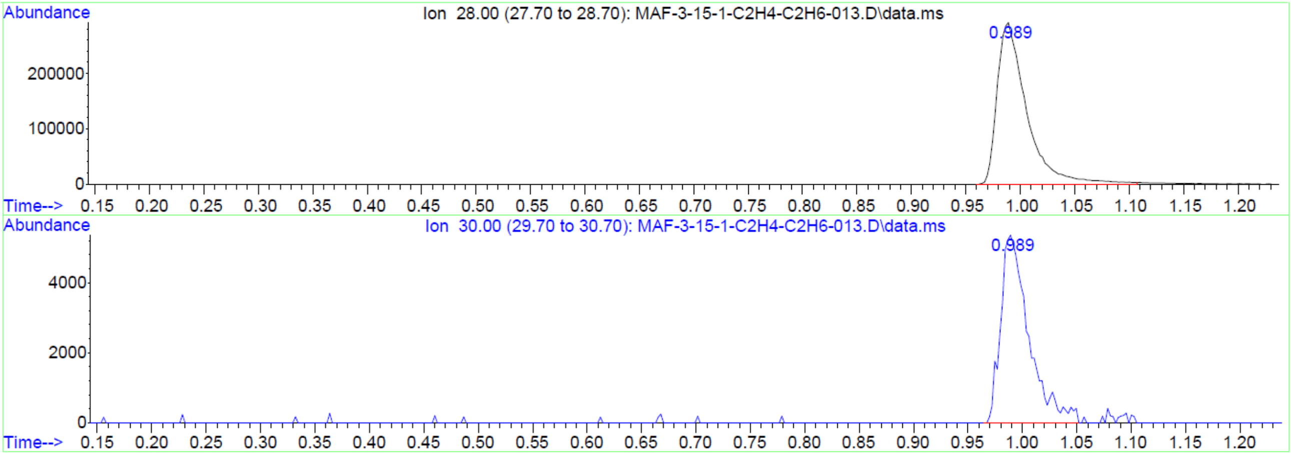

Supplementary Figure 22. GC-MS spectra of the outlet gas (top: C_2H_4, bottom C_2H_6) with C_2H_4 purity of 99.6% given by MAF-3 for a single breakthrough operation using 15:1 C_2H_4/C_2H_6 as input.

Area Percent Report

Data Path : H:\LiaoPQ-GC-MS-20150526-R2\
Data File : MAF-4-15-1-C2H4-C2H6-011.D
Acq On : 5 Apr 2015 8:57
Operator :
Sample :
Misc :
ALS Vial : 1 Sample Multiplier: 1

Integration Parameters: rteint.p
Integrator: RTE
Smoothing : ON Filtering: 5
Sampling : 1 Min Area: 3 % of largest Peak
Start Thrs: 0.2 Max Peaks: 100
Stop Thrs : 0 Peak Location: TOP

If leading or trailing edge < 100 prefer < Baseline drop else tangent >
Peak separation: 5

Method : E:\data\Jennifer\Flow throught system\GC methods\5PAHs_3Pesticides_liquid injection_splitless.M
Title :

Signal : EIC Ion 28.00 (27.70 to 28.70): MAF-4-15-1-C2H4-C2H6-011.D\data.ms

peak #	R.T. min	first scan	max scan	last scan	PK TY	peak height	corr. area	corr. % max.	% of total
1	0.995	347	359	449	rM 2	4881389	22194158	100.00%	100.000%

Sum of corrected areas: 22194158

Signal : EIC Ion 30.00 (29.70 to 30.70): MAF-4-15-1-C2H4-C2H6-011.D\data.ms

peak #	R.T. min	first scan	max scan	last scan	PK TY	peak height	corr. area	corr. % max.	% of total
1	0.997	349	360	383	rM 2	6935	12090	100.00%	100.000%

Sum of corrected areas: 12090

5PAHs_3Pest..._splitless.M Thu May 28 10:05:27 2015

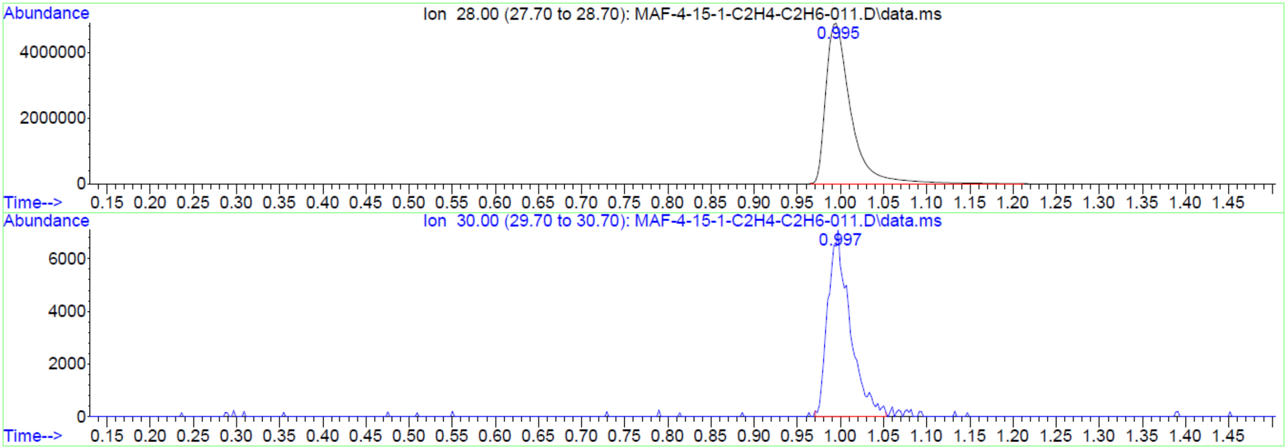

Supplementary Figure 23. GC-MS spectra of the outlet gas (top: C_2H_4, bottom C_2H_6) with C_2H_4 purity of 99.9% given by MAF-4 for a single breakthrough operation using 15:1 C_2H_4/C_2H_6 as input.

Area Percent Report

Data Path : H:\LiaoPQ-GC-MS-20150526-R2\
Data File : IRMOF-8-15-1-C2H4-C2H6-MS-009.D
Acq On : 31 Mar 2015 20:39
Operator :
Sample :
Misc :
ALS Vial : 1 Sample Multiplier: 1

Integration Parameters: rteint.p
Integrator: RTE
Smoothing : ON Filtering: 5
Sampling : 1 Min Area: 3 % of largest Peak
Start Thrs: 0.2 Max Peaks: 100
Stop Thrs : 0 Peak Location: TOP

If leading or trailing edge < 100 prefer < Baseline drop else tangent >
Peak separation: 5

Method : E:\data\Jennifer\Flow throught system\GC methods\5PAHs_3Pesticides_liquid injection_splitless.M
Title :

Signal : EIC Ion 28.00 (27.70 to 28.70): IRMOF-8-15-1-C2H4-C2H6-MS-009.D\data.ms

peak #	R.T. min	first scan	max scan	last scan	PK TY	peak height	corr. area	corr. % max.	% of total
1	0.929	319	332	410	rM 4	2778384	76935635	100.00%	100.000%

Sum of corrected areas: 76935635

Signal : EIC Ion 30.00 (29.70 to 30.70): IRMOF-8-15-1-C2H4-C2H6-MS-009.D\data.ms

peak #	R.T. min	first scan	max scan	last scan	PK TY	peak height	corr. area	corr. % max.	% of total
1	0.948	316	340	372	rM 4	12216	26104	100.00%	100.000%

Sum of corrected areas: 26104

5PAHs_3Pest..._splitless.M Thu May 28 10:07:29 2015

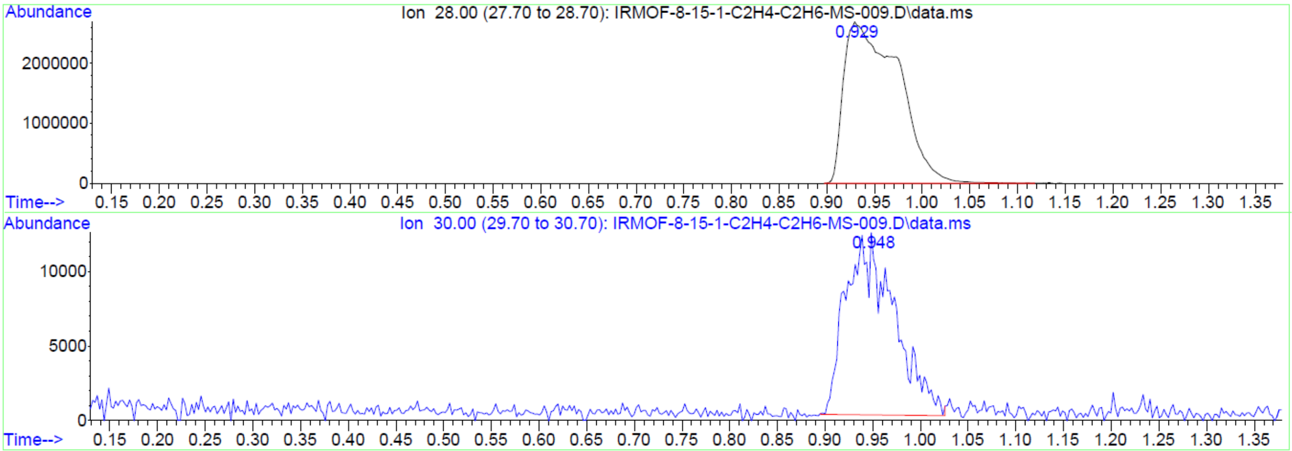

Supplementary Figure 24. GC-MS spectra of the outlet gas (top: C_2H_4, bottom C_2H_6) with C_2H_4 purity of 99.96% given by IRMOF-8 for a single breakthrough operation using 15:1 C_2H_4/C_2H_6 as input.

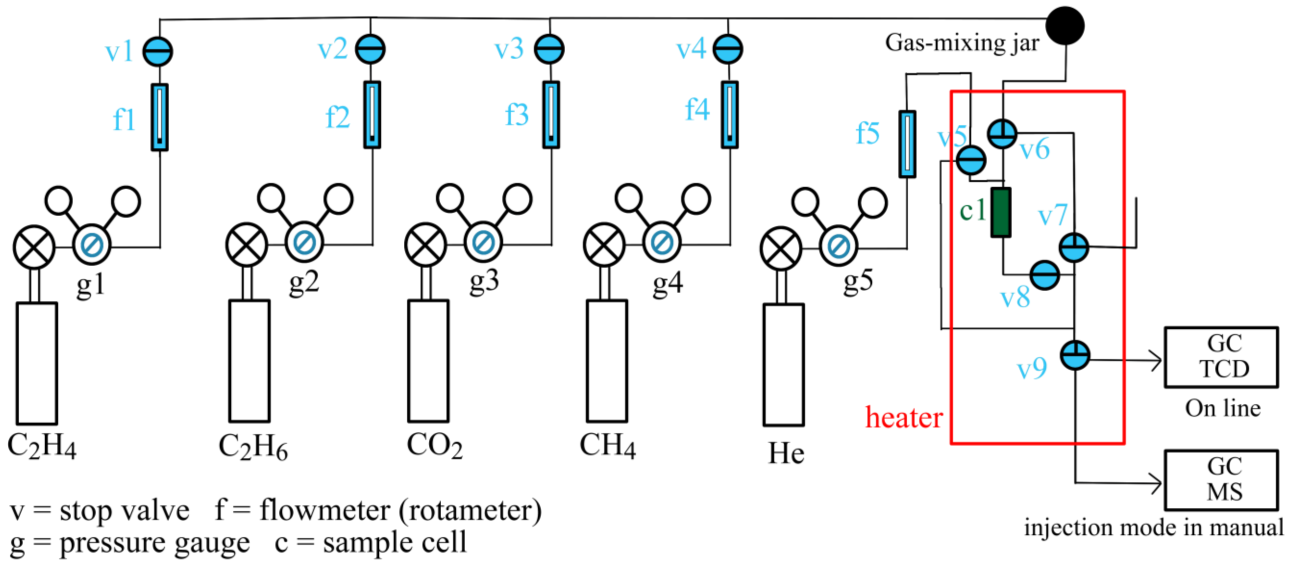

Supplementary Figure 25. The column breakthrough experiment setup.

Supplementary Table 1. Physical parameters of CO_2, C_2H_6 and CH_4[4, 5].

	CO_2	CH_4	C_2H_6	C_2H_4
Kinetic diameter (Å)	3.3	3.758	4.443	4.163
Polarizability ($\times10^{25}$ cm^3)	29.11	25.93	44.3-44.7	42.52
Quadruple moment ($\times10^{26}$ esu cm^2)	4.30	0	0.65	1.50
Boiling point (K)	194.75	111.6	184.55	169.42

Supplementary Table 2. Crystal Data and Structure Refinement results.

Complex	MAF-49	MAF-49·H_2O	MAF-49·C_2H_4	MAF-49·CO_2	MAF-49·C_2H_6
Formula	$C_5H_6N_8Zn$	$C_5H_7N_8O_{0.5}Zn$	$C_{5.78}H_{7.56}N_8Zn$	$C_{5.14}H_6N_8O_{0.27}Zn$	$C_{5.67}H_8N_8Zn$
Formula weight	243.57	252.56	254.50	249.56	253.62
Temperature (K)	195(2)	195(2)	195(2)	195(2)	195(2)
Space group	$P3_121$	$P3_121$	$P3_121$	$P3_121$	$P3_121$
a/Å	9.6963(15)	9.6767(14)	9.6896(16)	9.7682(15)	9.663(3)
c/Å	20.126(3)	20.172(3)	20.153(4)	20.100(4)	20.224(5)
V/Å^3	1638.7(4)	1635.8(4)	1638.6(5)	1660.9(5)	1635.2(8)
Z	6	6	6	6	6
D_c /g cm^{-3}	1.481	1.538	1.547	1.499	1.550
R_{int}	0.0192	0.0340	0.0254	0.0393	0.0529
R_1 [$I > 2\sigma(I)$]	0.0184	0.0369	0.0337	0.0422	0.0448
wR_2 (all data)	0.0438	0.0994	0.1028	0.1313	0.1203
GOF	1.060	1.051	1.059	1.052	1.008
Flack	0.003(10)	-0.02(2)	-0.01(2)	-0.01(3)	-0.02(3)
Peak and hole (e Å^{-3})	0.293 and -0.249	0.579 and -0.405	0.986 and -0.368	1.281 and -0.423	0.656 and -0.633

$R_1 = \sum||F_o| - |F_c||/\sum|F_o|$. $wR_2 = [\sum w(F_o^2 - F_c^2)^2/\sum w(F_o^2)^2]^{1/2}$.

Supplementary Table 3. Comparison for the binding energies between adsorption isotherms and GCMC/PDFT simulations.

Compound	From adsorption isotherms ($kJ\ mol^{-1}$)	From GCMC/PDFT simulation ($kJ\ mol^{-1}$)
MAF-49·CO_2	30	31
MAF-49·C_2H_4	48	47
MAF-49·C_2H_6	60	56

Supplementary Table 4. The interactions between the gas molecules and host framework in MAF-49.

C_2H_6				C_2H_4				CO_2			
	D···A (Å)	H···A (Å)	∠D-H···A (°)		D···A (Å)	H···A (Å)	∠D-H···A (°)		D···A (Å)	H···A (Å)	∠D-H···A (°)
C6-H61···N8	3.23	2.15	168					C6···N8	2.91		
C7-H71···N1A	3.47	2.40	162	C6-H61···N8	3. 58	2.54	161	C6···N8A	2.91		
C6-H62···N8A/N3A	2.86/3.38	2.15/2.70	120/120	C7-H71···N2A	3.57	2.65	142	C3-H31···O1	3.34	2.46	135
C6-H63···N2	4.15	3.26	140					C3A-H31A···O1A	3.34	2.46	135
C7-H72···N6B	4.59	3.59	152	C6-H62···N6	5.20	4.41	134				
C7-H73··N4C	5.05	4.03	155	C7-H71···N3	6.51	5.52	154				

Symmetric codes: A = 1+*x*-*y*, 2-*y*, 0.6667-*z*; B = 1+*x*-*y*, 1-*y*, 0.6667-*z*; C = -1-*x*+*y*, 1-*x*, -0.33333+*z*; D = *x*-*y*, 1-*y*, 0.6667-*z*.

Supplementary Table 5. Comparing the parameters of the breakthrough columns studied in this work.

Materials	Sample weight (g)	Crystal density (g cm^{-3})	Apparent density (g cm^{-3})	Column voidage	Column free space (cm^3)
MAF-49	1.873	1.481	1.128	0.238	0.395
IRMOF-8	1.063	0.896	0.640	0.285	0.474
MAF-4	1.316	1.067	0.793	0.257	0.427
MAF-3	1.472	1.241	0.887	0.285	0.473
HKUST-1	1.119	0.879	0.674	0.233	0.387
MOF-74-Mg	1.147	0.920	0.691	0.249	0.413
MOF-74-Co	1.374	1.170	0.827	0.293	0.485
UiO-66	1.531	1.198	0.922	0.230	0.382

Apparent density = Sample weight / Column volume

Column voidage = 1 - Sample weight / Crystal density / Column volume

Column free space = Column volume × Column voidage

Supplementary Table 6. Comparing the C_2H_4 productivities of C_2H_6 selective adsorbents for a single breakthrough operation using 1:1 C_2H_4/C_2H_6 as input.

		Gravimetric/Volumetric productivity (mmol g^{-1}/mmol cm^{-3}) with different purities		
Materials	Crystal density (g cm^{-3})	99.95%+	99.5%+	99%+
MAF-49	1.480	0.28/0.42	0.30/0.45	0.32/0.47
IRMOF-8	0.896	0/0	0.05/0.04	0.11/0.10
MAF-4	1.067	0/0	0.02/0.02	0.03/0.03
MAF-3	1.241	0/0	0.004/0.005	0.01/0.01

Supplementary Table 7. Comparing the C_2H_4 productivities of C_2H_6 selective adsorbents for a single breakthrough operation using 15:1 C_2H_4/C_2H_6 as input.

		Gravimetric/Volumetric productivity (mmol g^{-1}/mmol cm^{-3}) with different purities			
Materials	Crystal density (g cm^{-3})	99.995%+	99.95%+	99.5%+	99%+
MAF-49	1.480	1.48/2.17	1.68/2.48	2.07/2.97	2.46/3.70
IRMOF-8	0.896	0/0	0.06/0.05	0.15/0.13	0.22/0.21
MAF-4	1.067	0/0	0/0	0.004/0.004	0.015/0.016
MAF-3	1.241	0/0	0/0	0.001/0.001	0.006/0.008

Supplementary Methods.

Calculations of adsorption enthalpies

The coverage-dependent adsorption enthalpy profiles were calculated from the sorption data measured at 298, 307 and 316 K by the Virial fitting method and Clausius-Claperyron equation.

Virial fitting: A Virial-type expression was used (eq. 1), which is composed of parameters a_i and b_i, which are independent of temperature. In eq. 1, P is the pressure in atm, N is the adsorbed amount in mmol g^{-1}, T is the temperature in Kelvin, a_i and b_i are the Virial coefficients, and m and n represent the numbers of coefficients required to adequately describe the isotherms.

$$\ln p = \ln n + \frac{1}{T}\sum_{i=0}^{l} a_i n^i + \sum_{j=0}^{m} b_j n^j \qquad (1)$$

$$K_H = \exp(-b_0) \times \exp(\frac{-a_0}{T}) \qquad (2)$$

The adsorption enthalpy (ΔH) is obtained from the following equation derived from the Clausius-Clapeyron equation (eq. 3).

$$Q_{st} = -R\left[\frac{\partial \ln p}{\partial (1/\mathrm{T})}\right]_n = -R\sum_{i=0}^{l} a_i n^i \qquad (3)$$

Breakthrough measurements.

A stainless-steel column with a length of 10 cm and an internal diameter of 0.46 cm (V_{column} = 0.23×0.23×3.14×10.0 = 1.66 cm^3) was packed with microcrystalline sample. The sample in each column was compressed as much as possible to obtain the best separation performance, and column voidages are similar for different samples. As shown in the above scheme, the column was connected to the GC by stainless-steel pipes with a combined length of 40 cm and an internal diameter of 0.30 cm (V_{tube} = 0.150×0.150×3.14×40 = 2.84 cm^3). The column and most parts of the pipelines between the injection and sampling ports were placed in a temperature-controlling oven. The flow rates (cm^3 min^{-1} at 298.15 K and 1 bar) of pure gases were regulated by mass flow controllers. Before breakthrough experiments, the columns were heated at 323 K for 10 hours and then cooled to the measurement temperature of 313(1) K, during which pure He flow (2.0 cm^3 min^{-1}) was kept passing through the columns. Pure gases were mixed by passing through the gas-mixing jar and then used

directly. Except otherwise specified, the mixed gas flow rates are 0.8, 0.2, and 1.6 $cm^3\ min^{-1}$ for the breakthrough operations using 1:1:1:1 $CH_4/CO_2/C_2H_4/C_2H_6$, 1:1 C_2H_4/C_2H_6, and 15:1 C_2H_4/C_2H_6 as inputs, respectively. The gas stream at the outlet of the column was analyzed on line by using a chromatographic analyzer (Agilent 7890A) with a thermal conductivity detector (TCD) and an HP-AL/S column or an Agilent 5975C Mass Spectrometer (MS) with the injection mode in manual.

Absolute and relative concentrations of each gas at the inlet and outlet are expressed by molar ratio. To compare the performances of different materials unambiguously, the specific injection amount τ (mmol g^{-1}) was used as the abscissa, meaning that the breakthrough time (min) was not only divided by the adsorbent weight (g) but also multiplied by the flow rate of the injection gas (mmol min^{-1}). For comparison, breakthrough curves using breakthrough time (min) and specific breakthrough time (min g^{-1}) as abscissa were also provided in the supplementary information.

The breakthrough curves were expressed by

$$f(\tau)=\frac{C_o(\mathrm{j},\ \tau)}{C_i(\mathrm{j})} \qquad (4)$$

Where $C_o(\mathrm{j},\ \tau)$ is the TCD peak area of gas j at the outlet at τ and $C_i(\mathrm{j})$ is the TCD peak area of gas j at the inlet (not change with τ). The purity of gas j (*i.e.* the molar ratio of gas j in the outlet gas mixture containing n types of gases) can be expressed by

$$g(\tau)=\frac{C_o(\mathrm{j},\ \tau)}{\sum_{\mathrm{j}=1}^{\mathrm{n}} C_o(\mathrm{j},\ \tau)}\times 100\% \qquad (5)$$

To achieve a sufficient resolution for GC capillary column before TCD analyses, the outlet gas analytes need to be diluted by large amounts of protective gas He. Therefore, gas j could not be detected by TCD when the $g(\tau)$ is lower than about 1%. To obtain the accurate concentrations and purities for the C_2H_4/C_2H_6 breakthrough experiments, the MS peak areas of C_2H_4 and C_2H_6 were used, in which the C_2H_4 purity was expressed by

$$g(\tau)=\frac{M_o(C_2H_4,\ \tau)}{M_o(C_2H_4,\ \tau)+M_o(C_2H_6,\ \tau)/0.8}\times 100\% \qquad (6)$$

Where $M_o(C_2H_4,\ \tau)$ and $M_o(C_2H_6,\ \tau)$ are the MS peak areas of C_2H_4 and C_2H_6 at τ, respectively.

The C_2H_4 productivity (q) is defined by the breakthrough amount of C_2H_4, which is calculated by integration of the breakthrough curve $f(\tau)$ during a period from τ_1 to τ_2 where the C_2H_4 purity is higher than or equal to a threshold value p (such as 99.95%):

$$q = \frac{C_i(C_2H_4)}{C_i(C_2H_4) + C_i(C_2H_6)} \times (\int_{\tau_1}^{\tau_2} f(\tau) d\tau), \ (g(\tau) \geq p) \quad (7)$$

Computational Details.

All simulations/calculations were performed by the Materials Studio 5.5 package. The preferred sorption locations were searched through Grand Canonical Monte Carlo (GCMC) simulations by using the fixed loading task and Metropolis method in the sorption calculation module. For all the GCMC simulations, the simulation box was set with 8 (2 × 2 × 2) unit cells while all the frameworks and the gas molecules were described by the universal forcefield (UFF). Both the framework and the gas molecules were regarded as rigid model and the QEq partial charges and QEq charges were employed to the atoms of the framework and guest molecules, respectively. The cutoff distance was set to 18.5 Å for the Lennard-Jones (LJ) interactions, and the electrostatic interactions and the van der Waals interactions were handled using the Ewald and Atom based summation method, respectively. The loading steps, equilibration steps and the production steps were all set to 1.0×10^7. The saturation/maximum uptakes were modeled at 316 K using the fixed pressure task and Metropolis method with 2.0×10^7 equilibration steps, followed by 2.0×10^7 production steps for computing the ensemble averages.

All energies were calculated by the periodic density functional theory (PDFT) method by the Dmol^3 module. Before the energy calculations, full geometry optimizations with fixed cell parameters were performed to the host-guest systems based on the adsorption conformations obtained from GCMC simulation. The widely used generalized gradient approximation (GGA) with the Perdew-Burke-Ernzerhof (PBE) functional and the double numerical plus d-functions (DNP) basis set, TS for DFT-D correction as well as the Effective Core Potentials (ECP) were used. The energy, force and displacement convergence criterions were set as 1×10^{-5} Ha, 2×10^{-3} Ha and 5×10^{-3} Å, respectively. The energy balance is expressed by the following equations:

$$\Delta E_{\text{ads}} = E_{\text{host+guest}} - E_{\text{apohost}} - E_{\text{guest}} \quad (8)$$

$$\Delta E_{trans} = E_{host} - E_{apohost} \quad (9)$$

$$\Delta E_{fitting} = E_{host+guest} - E_{host} - E_{guest} \quad (10)$$

Where ΔE_{ads} is the adsorption enthalpy, ΔE_{trans} is the energy change of the host framework after adsorption of guest, $\Delta E_{fitting}$ is the interaction energy between the guest and the final host framework, and $E_{host+guest}$, E_{guest}, $E_{apohost}$ and E_{host} are the energies of the final host-guest system, the free guest, the guest-free host framework, and the transformed host framework after adsorption, respectively.

Supplementary References

1. Rowsell, J.L.C., Eckert, J. & Yaghi, O.M. Characterization of H2 Binding Sites in Prototypical Metal−Organic Frameworks by Inelastic Neutron Scattering. *J. Am. Chem. Soc.* **127**, 14904-14910 (2005).
2. Pires, J., Pinto, M.L. & Saini, V.K. Ethane Selective IRMOF-8 and Its Significance in Ethane–Ethylene Separation by Adsorption. *ACS Appl. Mater. Interfaces* **6**, 12093-12099 (2014).
3. Pillai, R.S., Pinto, M.L., Pires, J., Jorge, M. & Gomes, J.R.B. Understanding Gas Adsorption Selectivity in IRMOF-8 Using Molecular Simulation. *ACS Applied Materials & Interfaces* **7**, 624-637 (2015).
4. Li, J.-R., Kuppler, R.J. & Zhou, H.-C. Selective gas adsorption and separation in metal-organic frameworks. *Chem. Soc. Rev.* **38**, 1477-1504 (2009).
5. He, Y. et al. A microporous lanthanide-tricarboxylate framework with the potential for purification of natural gas. *Chem. Commun.* **48**, 10856-10858 (2012).
